# A polygenic risk score for Alzheimer’s disease constructed using *APOE*-region variants has stronger association than *APOE* alleles with mild cognitive impairment in Hispanic/Latino adults in the U.S.

**DOI:** 10.1186/s13195-023-01298-3

**Published:** 2023-08-30

**Authors:** Tamar Sofer, Nuzulul Kurniansyah, Einat Granot-Hershkovitz, Matthew O. Goodman, Wassim Tarraf, Iris Broce, Richard B. Lipton, Martha Daviglus, Melissa Lamar, Sylvia Wassertheil-Smoller, Jianwen Cai, Charles S. DeCarli, Hector M. Gonzalez, Myriam Fornage

**Affiliations:** 1 Division of Sleep and Circadian Disorders, Brigham and Women’s Hospital, Boston, MA USA; 2 Department of Medicine, Harvard Medical School, Boston, MA USA; 3 CardioVascular Institute, Beth Israel Deaconess Medical Center, Boston, MA USA; 4 Institute of Gerontology, Wayne State University, Detroit, MI USA; 5 Department of Neurosciences, University of California San Diego, San Diego, CA USA; 6 Albert Einstein College of Medicine, Bronx, NY USA; 7 Department of Medicine, Institute for Minority Health Research, University of Illinois at Chicago, Chicago, IL USA; 8 Rush Alzheimer’s Disease Research Center, Rush University Medical Center, Chicago, IL USA; 9 Department of Epidemiology & Population Health, Department of Pediatrics, Albert Einstein College of Medicine, Bronx, NY USA; 10 Department of Biostatistics, University of North Carolina at Chapel Hill, Chapel Hill, NC USA; 11 Department of Neurology, University of California at Davis, Sacramento, CA USA; 12 Shiley-Marcos Alzheimer’s Disease Center, University of California San Diego, La Jolla, CA USA; 13 Institute of Molecular Medicine, The University of Texas Health Science Center at Houston, Houston, TX USA

**Keywords:** Admixture, Diverse populations, Polygenic risk score, Mild cognitive impairment, Cognitive decline

## Abstract

**Introduction:**

Polygenic Risk Scores (PRSs) are summaries of genetic risk alleles for an outcome.

**Methods:**

We used summary statistics from five GWASs of AD to construct PRSs in 4,189 diverse Hispanics/Latinos (mean age 63 years) from the Study of Latinos-Investigation of Neurocognitive Aging (SOL-INCA). We assessed the PRS associations with MCI in the combined set of people and in diverse subgroups, and when including and excluding the *APOE* gene region. We also assessed PRS associations with MCI in an independent dataset from the Mass General Brigham Biobank.

**Results:**

A simple sum of 5 PRSs (“PRSsum”), each constructed based on a different AD GWAS, was associated with MCI (OR = 1.28, 95% CI [1.14, 1.41]) in a model adjusted for counts of the *APOE*-$\epsilon 2$ and *APOE*-$\epsilon 4$ alleles. Associations of single-GWAS PRSs were weaker. When removing SNPs from the *APOE* region from the PRSs, the association of PRSsum with MCI was weaker (OR = 1.17, 95% CI [1.04,1.31] with adjustment for *APOE* alleles). In all association analyses, *APOE*-$\epsilon 4$ and *APOE*-$\epsilon 2$ alleles were not associated with MCI.

**Discussion:**

A sum of AD PRSs is associated with MCI in Hispanic/Latino older adults. Despite no association of *APOE*-$\epsilon 4$ and *APOE*-$\epsilon 2$ alleles with MCI, the association of the AD PRS with MCI is stronger when including the *APOE* region. Thus, *APOE* variants different than the classic *APOE* alleles may be important predictors of MCI in Hispanic/Latino adults.

**Supplementary Information:**

The online version contains supplementary material available at 10.1186/s13195-023-01298-3.

## Introduction

Hispanic/Latino people are the largest growing minority in the U.S., projected to represent 28.6% of the U.S. population by 2060 [1]. Rates of Alzheimer’s disease and related dementia (ADRD) and mild cognitive impairment (MCI), which often precede ADRD, are higher in Hispanics/Latinos compared to European Americans [2–4]. However, the strongest known genetic risk factor for ADRD, the *APOE*-$\epsilon 4$ allele [5], has weaker association in Hispanics/Latinos compared to individuals of European ancestry [6], and was not associated with MCI in recent studies from the Study of Latinos – Investigation of Cognitive Aging (SOL-INCA) [7, 8]. Polygenic Risk Scores (PRSs) are aggregated summaries of genetic data, generally defined as weighted sums of counts of alleles associated with a particular health outcome across the genome. Thus, by collecting information genome-wide, PRSs may assist in explaining the genetic association of ADRD and MCI beyond the *APOE* alleles and perhaps help elucidate ADRD disparities in Hispanics/Latinos to some extent. Further, as PRS are more developed, they are starting to become useful for risk prediction [9], potentially leading to disease prevention [10], e.g. by risk stratification, and by personalizing interventions [11]. Thus, applying PRS to evaluate personalized susceptibility for ADRD and MCI may be a useful target that will ultimately improve these outcomes among Hispanic/Latino adults.

PRSs are typically constructed based on summary statistics from genome-wide association studies (GWAS). It is already known that, to be useful for PRS construction, a GWAS needs to have a large enough sample size [12]. By now, published GWAS of AD are available from a few studies and large consortia, with the largest GWAS based on European ancestry individuals, but others including multi-ethnic and African populations [13–18]. Hispanics/Latinos are admixed, with European, African, and Amerindian ancestries, with varying degrees of admixture across groups defined by Hispanic/Latino background [19]. While no large GWAS matches the genetic ancestry composition of the SOL-INCA Hispanic/Latino individuals exactly, in multiple GWAS in the Hispanic Community Health Study/Study of Latinos (HCHS/SOL), we showed that many genetic loci for cardiometabolic and other complex traits identified in GWAS of other genetic ancestries also show associations in Hispanics/Latinos [20–24]. Earlier studies of PRSs that were typically developed based on summary statistics from GWAS of smaller sample sizes than recent GWAS did not result in high transferability to Hispanic/Latino populations [25]. However in recent studies including Hispanic/Latino individuals from HCHS/SOL and other studies from the Trans-Omics in Precision Medicine (TOPMed) initiative, we saw improved PRS performance in Hispanic/Latino individuals, even when PRSs were developed based on GWAS of European ancestry, with multi-ethnic GWAS further improving polygenic models performance [26–28]. Thus, PRS constructed based on non-Latino GWAS of AD has the potential to predict AD or MCI in Hispanic/Latino adults.

The SOL-INCA is an ancillary study to the HCHS/SOL [29], designed to study the development of ADRD in U.S. Hispanics/Latinos. The average age at the SOL-INCA exam was 62, allowing for assessment of MCI, but not yet of ADRD, with MCI being defined using the National Institute on Aging– Alzheimer’s Association criteria for MCI syndromes [30]. While we do not know yet how MCI will predict future ADRD, it is important to study how known genetic factors underlying ADRD may predict MCI in this population. Recently, Logue et al. [31] reported an association of a PRS constructed based on a GWAS of AD from IGAP (international genomics of Alzheimer’s project; European ancestry individuals) [16] with MCI in a sample of middle aged (mean age 56) Americans of European ancestry. It remains to study whether AD PRS, constructed based on GWAS in European ancestry individuals or in a multi-ethnic analysis, is associated with MCI in Hispanics/Latinos and whether *APOE* alleles impact this association.

**Fig. 1:**
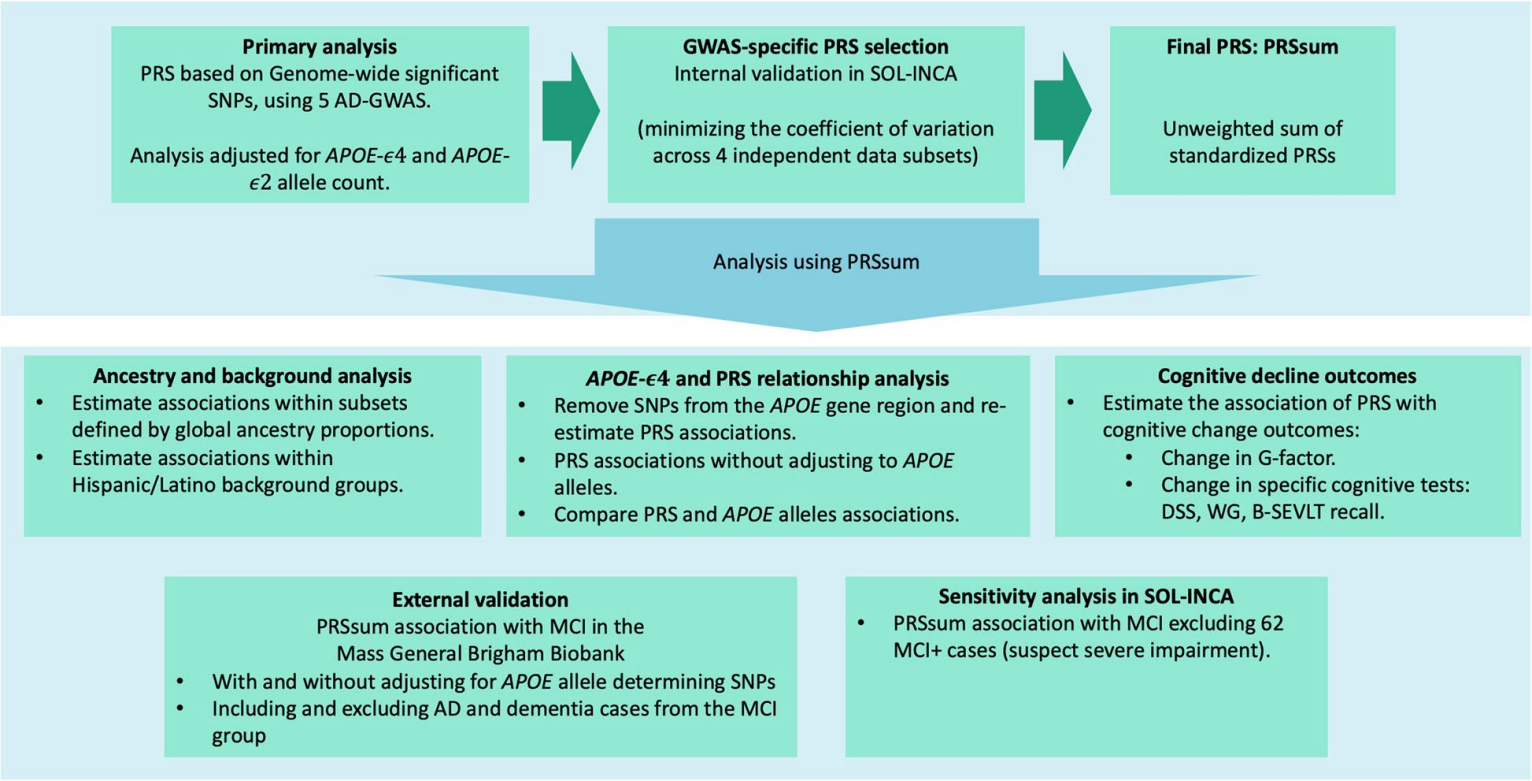
Analysis flowchart

Here, we use summary statistics from five GWAS of Alzheimer’s disease to develop PRSs with and without inclusion of single nucleotide polymorphisms (SNPs) from the *APOE* gene region. Based on each GWAS, we train PRSs using multiple tuning parameters and select the one PRS that has the strongest potential to predict MCI based on an internal model validation across independent subsets of the SOL-INCA dataset. We further combine the PRSs in an unweighted sum (applied on standardized PRSs) called PRSsum, following previous work [26]. The idea behind sum of PRSs is to combine information that is captured in difference ways by different GWAS, due to differences in their study populations. While different PRSs may represent, to some extent, the same genomic regions, because they are standardized the overall contribution of a given genomic region may not be overly amplified but rather represents a weighted combination of its contribution to the various PRSs. We estimate the associations of these PRSs with MCI, and examine whether the associations depend on genetic ancestry and the *APOE* genotypes by including and excluding *APOE* gene-region variants from the PRSs. We also investigate the association of the PRSs with change in global cognitive function and in specific domains. Finally, we report the association of these PRSs with MCI in the Mass General Brigham (MGB) Biobank dataset. The conceptual organization of this study is described in Fig. 1.

## Methods

### The Hispanic Community Health Study/Study of Latinos

The HCHS/SOL [32–34] is a population-based longitudinal cohort following Hispanic/Latino participants from four metropolitan areas: Bronx NY, Miami FL, Chicago IL, and San Diego CA, with 16,415 participants aged 18–74 years examined in the baseline visit. Participants self-identified with six Hispanic/Latino background groups: Central American, South American, Mexican (Mainland groups, have high Amerindian genetic ancestry and low African ancestry), Cuban (high proportion of European ancestry, low African and Amerindian ancestry proportions) Dominican, and Puerto-Rican (Caribbean group, have low Amerindian ancestry, and high African ancestry proportions). At baseline, participants who were at least 45 years old and did not refuse nor had health limitations (*n* = 9,714) were administered cognitive tests [35]. A second clinic visit occurred in 2014–2017, and during or after this visit, 6,377 participants who were eligible (completed neurocognitive testing during visit 1 and were at least 50 years old at visit 2) participated in the SOL-INCA), an ancillary study to the HCHS/SOL. SOL-INCA exams occurred, on average, 7 years after the baseline visit. Our primary phenotype was MCI, defined according to the National Institute on Aging-Alzheimer’s Association (NIA-AA) criteria [30]. Detailed information about the SOL-INCA exam and cognitive phenotyping is available in [29]. In this study, we included *n* = 4,256 individuals who participated in both the SOL-INCA study and were genotyped. All individuals provided written informed consent at their recruitment site. Additional information about the HCHS/SOL, SOL-INCA, cognitive phenotypes, and genotyping and imputation, is provided in the Supplementary Information.

### Discovery GWAS of Alzheimer’s disease

We used summary statistics from three publicly available GWAS of AD, as well as two GWAS that required application to the NIAGADS database [36]. These included three GWAS of European ancestry populations, including from the FinnGen Biobank and a GWAS incorporating an AD-by-proxy analysis [14, 17, 37], a GWAS of populations of African descent [13], and a multi-ethnic GWAS [15].

### PRS construction

Genotyping and imputations are described in the Supplementary Information. We constructed a range of PRSs based on the GWAS listed in Table 1 using two approaches: the clump-and-threshold method implemented in the PRSice 2 software [38] with HCHS/SOL as the reference panel, and using one of the modern Bayesian methods, PRS-CS (auto) software [39] or LDPred2 (auto) [40], implemented in the R package bigsnpr [41]. We used PRS-CS with UK-Biobank based reference panel matching the ancestry of the population used for GWAS for each ancestry-specific PRS, and LDpred2 when using summary statistics from multi-population GWAS. In this case we used the HCHS/SOL genotyping dataset as the LD reference panel, as there is no reference LD panel that exactly matches the GWAS combined population. We first lifted over summary statistics from genome build 37 to genome build 38 (other than for two GWAS already using hg38), and removed summary statistics corresponding to SNPs with minor allele frequency lower than 1%, where allele frequency was computed based on the HCHS/SOL dataset. For PRSs that use clumped SNPs, we applied PRSice to create PRS using clumping parameters *R*^2^ $\in \left\{0.1, 0.2, 0.3\right\}$ and distances of 250 Kb, 500 Kb, and 100 Kb. For a given set of *R*^2^ and distance clumping parameters, this means that once a SNP is selected, all other SNPs within that distance and with correlation higher than the set *R*^2^ are removed from consideration. *P*-value thresholds used by PRSice on the summary statistics were: $\{5\times {10}^{-8}, {10}^{-7}, {10}^{-6}, {10}^{-5}, {10}^{-4},{10}^{-3}, {10}^{-2}, 0.1, 0.2, 0.3, 0.4, 0.5\}$. Thus, for a given p-value threshold, there are multiple PRSs constructed, corresponding to the various clumping parameters. Because we do not have access to a similar Hispanic/Latino population with MCI, we followed a previous manuscript [26] and used an internal validation approach for selecting the best performing PRS: we split the SOL-INCA dataset into 4 random, distinct, sets of genetically-unrelated individuals, and estimated the association of each of the PRSs with MCI (as described below). The selected PRS from each GWAS was the one that minimized the coefficient of variation computed over the 4 estimated effect sizes (log odds-ratio). Finally, we also constructed PRSsum as an unweighted sum of the selected PRS from all considered GWAS. PRSs were summed without weights, after scaling them to have mean zero and standard deviation of 1. We did not develop a weighted sum of PRSs due to lack of an appropriate, external, dataset for training weights.

**Table 1:** External GWAS used for AD PRS construction

GWAS name	Sample size (cases/controls)	Outcome	Race/ethnicity	Reference	Data download link
Kunkle et al. 2019 [17]	21,982 cases 41,944 controls (from stage 1)	AD	European ancestry	PMID: 30820047	https://www.niagads.org/datasets/ng00075
Kunkle et al., 2021 [13]	2,784 cases and 5,222 controls	AD	African ancestry	PMID: 33074286	https://www.niagads.org/datasets/ng00100
Bellenguez et al. [14]	39,106 cases, 46,828 proxy-AD cases and 401,577 controls	AD	European ancestry	PMID: 35379992	https://www.ebi.ac.uk/gwas/studies/GCST90027158
FINNGEN	7,759 cases, 334,740 controls	AD	Finnish European		https://www.FINNGEN.fi/en/access_results We used R8 GWAS version published on Dec, 1 2022
Jun et al. [15]	15,579 cases, 17,690 controls	AD	Multi-ethnic: European ancestry, African American, Japanese, and Israeli Arab	PMID: 28183528	https://www.niagads.org/datasets/ng00056
Lake et al. [42]	54,233 cases, 46,828 proxy-AD cases and 543,127	AD	Multi-ethnic: European ancestry, Finish European, East Asian, African ancestry, Caribbean Hispanic	PMID: 37198259	https://ndkp.hugeamp.org/dinspector.html?dataset=Lake2023_AD_Mixed

In another analysis, we removed SNPs from the *APOE* region, defined here as 1 Mb region centered at chr19:44908822 (hg38) from the selected PRSs. The corresponding PRSsum was computed over the PRSs without *APOE* region SNPs.

We also benchmarked the selected PRS against a PRS constructed using only the lead variants from the combined stage 1 and stage 2 analysis of Bellenguez et al. [14], and a newly published AD GWAS from Lake et al. (2023) [42], where for the latter we also selected the “best PRS” by minimizing the CV. When constructing PRS based on Lake et al. GWAS, we used summary statistics from their random effects meta-analysis.

### PRS association analysis in SOL-INCA

In primary analysis, we used the combined SOL-INCA population. PRS were standardized in association testing so that they had mean zero and variance 1, and estimated effect sizes are per 1 SD of the PRS. Standardizations were performed on the combined SOL-INCA population and were not performed again when considering subgroups. The association analyses used logistic (for MCI) and linear (for cognitive decline phenotypes) mixed models implemented in the GENESIS R package [43], adjusted to sex, age at the baseline cognitive exam, time between the baseline exam and the SOL-INCA exam, education level (3-category variable: less than higher school diploma or GED, high school diploma or GED, or higher), study center, 5 principal components of genetic data, and for APOE-$\epsilon 4$ and APOE-$\epsilon 2$ allele counts, and with random effects corresponding to kinship, household, and block unit sharing. In a sensitivity analysis, we removed 62 individuals with MCI + (suspect severe cognitive deficit) and re-evaluated the PRS associations. Focusing on the primary PRSsum, we also performed additional analyses: we estimated PRS associations across Hispanic/Latino background groups and groups defined by having at least 20% of a given genetic ancestry (European, African, Amerindian), and PRS associations with cognitive decline phenotypes.

To assess whether the best performing PRS had statistically stronger association with MCI compared to other PRSs, we used the r2redux R package [44], that implements a method to test the difference between the prediction performance of a pair of PRSs. For this, we used the subset of unrelated individuals.

### Mass General Brigham Biobank and PRS validation

As a form of validation of the association of AD PRS with MCI in an external dataset, we constructed the selected PRSs in the Mass General Brigham (MGB) Biobank, a biorepository of consented patient samples at the MGB healthcare institutions. We queried the MGB Biobank portal on December 20, 2022, and restricted the query to individuals with genetic data who are at least 50 years old, so that MCI is more likely to be aging-related, and further, this minimum age matched that of SOL-INCA participants. We extracted MCI using the term “Mild cognitive impairment- so stated” and AD and dementia status using the terms “Alzheimer’s disease/Dementia”, “Alzheimer’s disease”, “Arteriosclerosis dementia”, and “Lewy body dementia” and assumed that participants had this status in their last encounter in the system (i.e. their current age, or most recent age if they are deceased). MCI cases were defined as individuals with MCI, and controls were individuals without MCI and without AD or dementia status. Genetic data were imputed to the TOPMed reference panel. Genotyping and imputation are described in the Supplementary Information. The PRSs selected based on HCHS/SOL analysis were constructed using the PRSice2 package, without any further clumping or thresholding. We used unrelated individuals (3^rd^ degree, identified using PLINK). To allow for potential comparison with SOL-INCA, we used the estimated means and standard deviations of the PRSs from SOL-INCA to standardize the PRSs in the MGB Biobank. Association analyses between PRSs and MCI were performed using logistic models and adjusted for age, sex, genotyping batch, with and without *APOE* SNPs, and 10 genetic PCs. *APOE* alleles were not available for everyone in the dataset, hence we used the two SNPs determining the *APOE* alleles instead. To assess whether the PRS associations with MCI are due to AD and dementia, we performed analysis in which we allowed for, and analysis in which we excluded, AD and dementia cases in the MCI group. For associations that were null in the MGB Biobank, we performed power analysis using the powerMediation R package version 0.3.4 (function powerLogisticCon) to assess whether the null result is likely due to low power.

## Results

Table 2 characterizes the target population of the SOL-INCA study, by Hispanic/Latino background group. At the SOL-INCA exam, the average age ranged from 62–65 years in the target population across Hispanic/Latino background groups, with the Cuban group being oldest on average with mean age 65.2. In many other characteristics, such as education, rates of MCI, and global proportions of genetic ancestries, the background groups were quite heterogeneous.

**Table 2:** Target population characteristics by Hispanic/Latino background group (survey weighted)

Characteristics	Hispanic/Latino Background					
	**Central American**	**Cuban**	**Dominican**	**Mexican**	**Puerto Rican**	**South American**
N	457	939	446	1583	820	344
Age at SOL-INCA visit (mean (sd))	62.30 (7.40)	65.20 (8.57)	62.81 (8.18)	62.04 (7.58)	63.97 (8.13)	63.38 (7.98)
Sex = M (%)	41.60	52.20	40.20	46.60	48.10	43.10
BMI (mean (sd))	30.65 (5.68)	29.63 (5.35)	30.14 (5.18)	30.07 (5.30)	30.84 (6.29)	29.18 (5.12)
Education (%)						
• No high school diploma	39.40	24.10	43.60	46.20	43.30	18.50
• At most a High school diploma	19.50	24.50	19.70	21.00	20.50	20.21
• Greater than high school	41.10	51.40	36.70	32.80	36.20	61.50
Hypertension = Yes (%)	38.30	56.30	57.20	37.20	52.80	33.60
Type 2 Diabetes = Yes (%)	28.40	27.80	29.70	27.80	32.60	21.40
APOE-e4 allele carrier = Yes (%)	21.20	23.50	29.20	19.50	23.40	16.20
APOE-e2 allele carrier = Yes (%)	7.40	11.6	18.80	5.10	9.70	5.50
Prop of European ancestry (mean (SD))	0.46 (0.15)	0.80 (0.21)	0.48 (0.15)	0.46 (0.20)	0.65 (0.12)	0.50 (0.22)
MCI = Yes (%)	11.70	11.00	12.60	10.40	12.80	8.00
G-Factor change (mean (SD))	-0.09 (0.85)	-0.08 (0.97)	-0.08 (0.85)	-0.21 (0.84)	-0.18 (0.85)	-0.07 (0.93)
Word frequency change (mean (SD))	-0.56 (5.34)	-0.28 (5.74)	0.52 (5.57)	-0.93 (5.44)	0.15 (5.21)	-0.75 (5.76)
SEVLT recall change (mean (SD))	0.28 (2.95)	0.28 (3.01)	-0.13 (2.89)	-0.10 (2.87)	-0.26 (2.93)	-0.01 (3.16)
Digit symbol change (mean (SD))	-2.22 (7.41)	-2.22 (8.28)	-2.06 (6.93)	-2.51 (6.84)	-2.78 (7.93)	-0.26 (7.35)

The table characterizes the target population represented by the study participants. All means, standard deviations (SDs), and percentages, were computed while accounting for survey design

### PRS associations with MCI

**Fig. 2:**
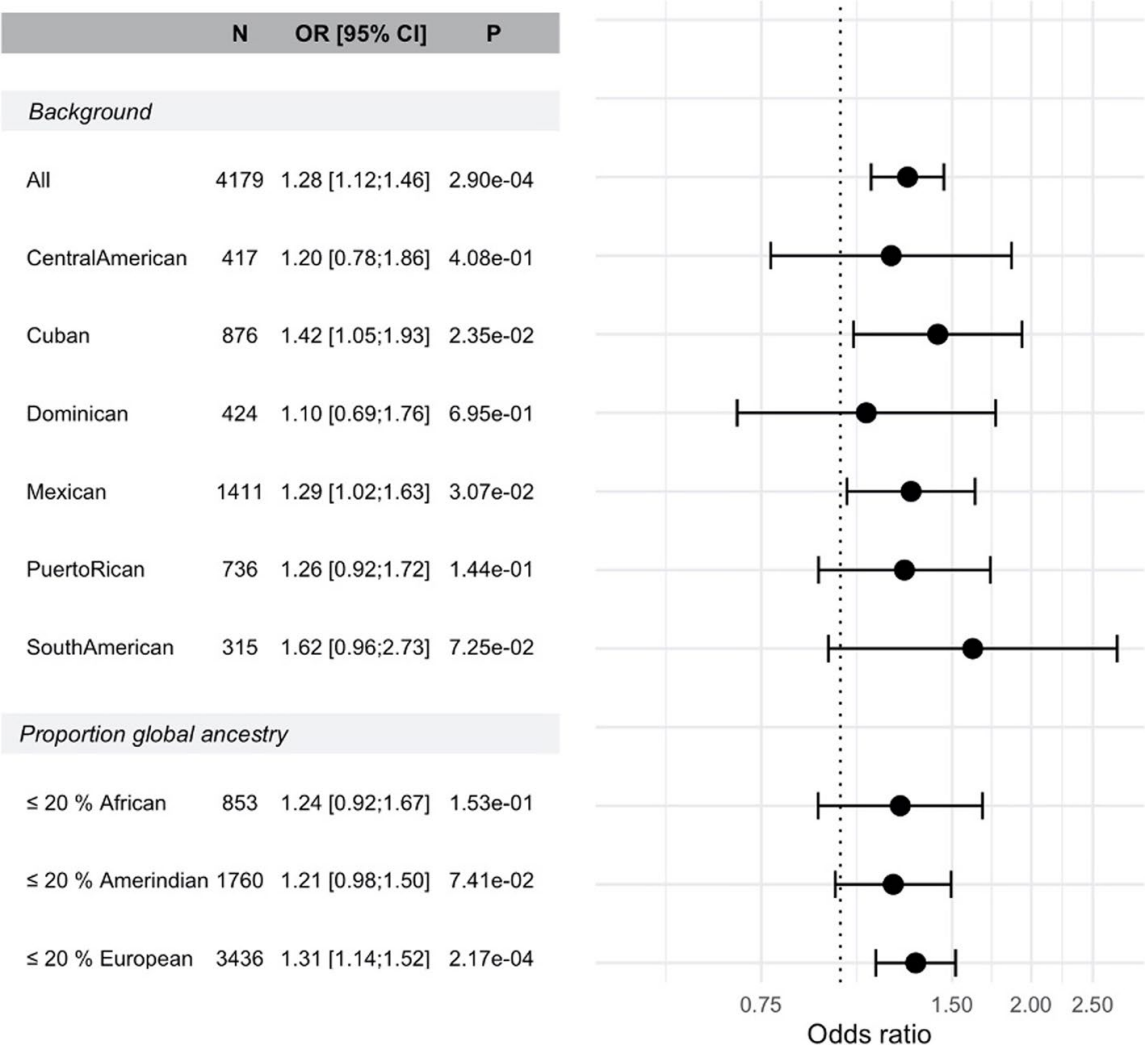
Estimated effect sizes and confidence intervals of PRSsum based on AD GWASs in association with MCI. PRSsum was constructed as the unweighted sum of 5 standardized PRSs, each based on a separate GWAS described in Table 1. For each GWAS, the PRSs were selected based on optimizing the coefficient of variation across 4 independent subsets of the SOL-INCA dataset. We provide the estimated effect size (odds ratio), 95% confidence interval, and *p*-value (computed based on the Score test) in models based on the complete dataset (“All”), by Hispanic/Latino background, and for the subsets of people with at least 20% global proportion of African, Amerindian, and European ancestries. The PRS associations were estimated in models adjusted for age at the HCHS/SOL baseline visit, time from HCHS/SOL baseline to the SOL-INCA visit, sex, study center, 5 principal components, and APOE-$\epsilon 4$ and APOE-$\epsilon 2$ allele counts

Based on each GWAS, we selected a single PRS that minimized the coefficient of variation computed across PRS estimated effect sizes from 4 independent subsets of the analytic sample. All selected PRSs were developed using the clumping & thresholding methodology in PRSice 2. Supplementary Table 1 provides the clumping and threshold parameters for the selected PRSs, and Supplementary Table 2 provides the attained CVs of PRSs constructed using the Bayesian methods PRS-CS and LDPred2, demonstrating that they are higher than the CVs of PRSice-based PRSs. The final PRS is PRSsum, which sums without weights the five GWAS-based PRSs after standardizing them to have mean 0 and variance 1. Table 3 provides all association analysis results for the five individual PRSs and PRSsum in association with MCI, in models with and without adjusting for *APOE* alleles, and for PRSs excluding *APOE* region SNPs. Figure 2 describes the association of PRSsum with MCI in terms of estimated odd ratios (OR) and 95% confidence intervals, for MCI in the combined SOL-INCA dataset, and within restricted subsets of Hispanic/Latino background, and of participants defined by having at least 20% global European, African, or Amerindian genetic ancestry. Supplementary Figs. 1–5 provide the corresponding figures for each of the individual GWAS PRSs. While PRSsum had better performance than each of the component PRSs, we tested the difference in prediction performance (as measured by R^2^) between PRSsum and each of its component PRSs. The results are reported in Supplementary Table 3. The prediction differences between PRSsum and the other PRSs were not statistically significant.

**Table 3:** Estimated associations of AD PRS and of *APOE *allele counts with MCI

Study	GWAS Population	#SNP	*APOE-ϵ2*	P	*APOE-ϵ4*	P	PRS	P
PRS includes *APOE* region SNPs, analysis unadjusted for *APOE* alleles								
FinnGen	Finish	81	–	–	–	–	1.17[1.06;1.29]	2.00E-03
Jun et al	Multi-Ethnic	85	–	–	–	–	1.10[1.00;1.21]	0.05
Bellenguez et al	White	1937	–	–	–	–	1.07[0.97;1.18]	0.17
Kunkle et al. 2019 [17]	White	12002	–	–	–	–	1.27[1.01;1.61]	0.04
Kunkle et al. 2021 [13]	Black	157	–	–	–	–	1.11[1.01;1.22]	0.03
PRSsum	Multi-Ethnic	14100	–	–	–	–	1.19[1.07;1,32]	9.16E-04
PRS includes *APOE* region SNPs, analysis adjusted for *APOE* alleles								
FinnGen	Finish	81	1.02[0.71;1.47]	0.91	0.95[0.73;1.22]	0.67	1.19[1.06;1.33]	3.00E-03
Jun et al	Multi-Ethnic	85	0.95[0.66;1.36]	0.78	0.89[0.65;1.21]	0.46	1.15[1.01;1.31]	0.04
Bellenguez et al	White	1937	0.98[0.68;1.40]	0.89	1.09[0.85;1.38]	0.5	1.06[0.96;1.18]	0.27
Kunkle et al. 2019 [17]	White	12002	0.98[0.68;1.41]	0.91	1.06[0.84;1.35]	0.61	1.34[1.05;1.71]	0.02
Kunkle et al. 2021 [13]	Black	157	0.95[0.66;1.36]	0.77	0.96[0.74;1.25]	0.75	1.14[1.02;1.28]	0.02
PRSsum	Multi-Ethnic	14100	1.02[0.71;1.46]	0.92	0.81[0.61;1.09]	0.16	1.28[1.12;1.46]	2.00E-04
PRS excludes *APOE* region SNPs, analysis adjusted for *APOE* alleles								
FinnGen	Finish	34	0.96[0.67;1.38]	0.83	1.08[0.85;1.38]	0.51	1.14[1.03;1.27]	0.02
Jun et al	Multi-Ethnic	38	0.95[0.67;1.36]	0.79	1.10[0.86;1.39]	0.45	1.04[0.94;1.15]	0.48
Bellenguez et al	White	1871	0.96[0.67;1.37]	0.8	1.10[0.86;1.39]	0.45	1.03[0.93;1.14]	0.62
Kunkle et al. 2019 [17]	White	11805	0.96[0.67;1.37]	0.81	1.10[0.86;1.39]	0.45	1.27[0.99;1.63]	0.06
Kunkle et al. 2021 [13]	Black	146	0.95[0.67;1.36]	0.79	1.10[0.86;1.39]	0.45	1.11[1.00;1.22]	0.05
PRSsum	Multi-Ethnic	13807	0.96[0.67;1.38]	0.82	1.09[0.86;1.39]	0.46	1.17[1.04;1.31]	0.01

For each PRS, we estimated its association as well as the *APOE*-$\epsilon 4$ and *APOE*-$\epsilon 2$ allele counts associations with MCI in a joint model accounting for these variables together, and further adjusting for age at HCHS/SOL baseline visit, time between the baseline and SOL-INCA visit, sex, study center, and 5 first principal components of the genetic data. In the top part of the table the PRSs includes *APOE*-region SNPs and the analysis is not adjusted for *APOE* allele counts. In the middle part, the PRS associations are adjusted for APOE allele counts. At the bottom, *APOE*-region SNPs were excluded from the PRSs in analyses that do not adjust for APOE alleles

We also computed PRSs based on (1) the lead SNPs from Bellenguez et al. GWAS, and (2) the recently-published Lake et al. GWAS. Supplementary Figs. 6–7 provide results for these PRSs. The Bellenguez et al.-based PRSs using only lead SNPs performed slightly worse than the Bellenguez-based PRS selected and reported in Table 2. The Lake et al.-based PRS performed worse than most other single-GWAS PRSs.

As shown in Fig. 2, PRSsum were associated with MCI in the complete sample: OR = 1.28, 95% CI [1.14, 1.41], *p*-value = 0.0002). All PRSs other than that based on Bellenguez et al. were associated with MCI (Table 3). When removing 62 individuals who fell in a diagnostically unclear “gray zone” between MCI and dementia, the results were essentially the same (Supplementary Table 4). Figure 2 further demonstrates that when stratifying by Hispanic/Latino background, and when restricting to sets of individuals defined by with at least 20% of a given ancestry, the estimated ORs are similar, and the OR based on the combined population is withing the confidence intervals of all subgroup-specific estimates. Considering the PRSs based on individual GWASs (Supplementary Figs. 1–5) it is difficult to summarize results into a specific pattern, perhaps because of the low sample sizes under stratification.

### PRS associations with MCI in MGB Biobank

Supplementary Table 5 characterizes the MGB Biobank study population. There were 24,818 MGB Biobank individuals 50 or older. After excluding 1,660 individuals without MCI code but having AD or dementia code, 23,158 individuals remained in the dataset. There were 885 (3.8%) individuals with MCI, of which 320 (1.3%) had AD or dementia. Association analyses results are provided in Supplementary Table 6. In association analysis of PRSsum where the MCI group included AD and dementia cases, and the two SNPs defining the *APOE* alleles were used as covariates, PRSsum was associated with MCI with OR = 1.06 and *p*-value = 0.2. Only the PRS based on Bellenguez et al. had statistically significant association with MCI, with OR = 1.13 and *p*-value = 0.004. When excluding AD and dementia cases from the MCI group, none of the associations had *p*-value < 0.05. In analyses in which *APOE* alleles were removed from the regression model, and the MCI group included AD and dementia cases, all AD PRS had strong association with MCI, with PRSsum having the strongest association (OR = 1.27,*p*-value = 1 × 10^–15^). However, once removing AD and dementia cases from the MCI group, again all associations weakened, with only Bellenguez-based PRS having *p*-value < 0.05 (= 0.04). We performed power analysis to evaluate whether the null effects are due to the reduction in the number of cases. Given the sample size, proportion of MCI case, and effect size estimates either from HCHS/SOL analysis with and without *APOE* allele adjustment, or from MGB analysis when including AD cases, the power was always > 0.98, suggesting the these null results are not due to limited statistical power.

### Relationship between *APOE* and AD PRS

Table 3 reports the association of the AD PRS and of *APOE*-$\epsilon 4$ and *APOE*-$\epsilon 2$ allele counts, in a model that accounted for all these genetic components together, with MCI. It is noticeable that *APOE* alleles are not associated with MCI, while AD PRSs are more strongly associated with MCI when they include *APOE*-region SNPs. For example, the OR of PRSsum reduces from 1.28 (including *APOE*-region SNPs) to 1.17 (excluding *APOE*-region SNPs), and its p-value increases from 0.0002 to 0.01. The same pattern is observed or the individual GWAS PRSs. When using the primary PRSsum (including *APOE*-region SNPs) in an association model without *APOE* alleles, its association with MCI slightly weakens (OR = 1.19, *p*-value = 0.0009). The association of individual GWAS PRSs with MCI also slightly change, suggesting that all PRS are somewhat associated with *APOE* alleles. Supplementary Fig. 8 demonstrates that PRS distributions differ between carriers (having at least one) and non-carriers of the *APOE-*$\epsilon$* 4* allele. However, this difference is small for PRS based on Bellenguez et al. To address the possibility that differences in PRS distribution by *APOE-*$\epsilon$* 4* carrier status are driven by different ancestral genetic make-up, Supplementary Fig. 9 displays similar distributions limited to individuals with high proportion (> 80%) of European ancestry, demonstrating similar patterns.

### PRSsum associations with cognitive change outcomes

**Fig. 3:**
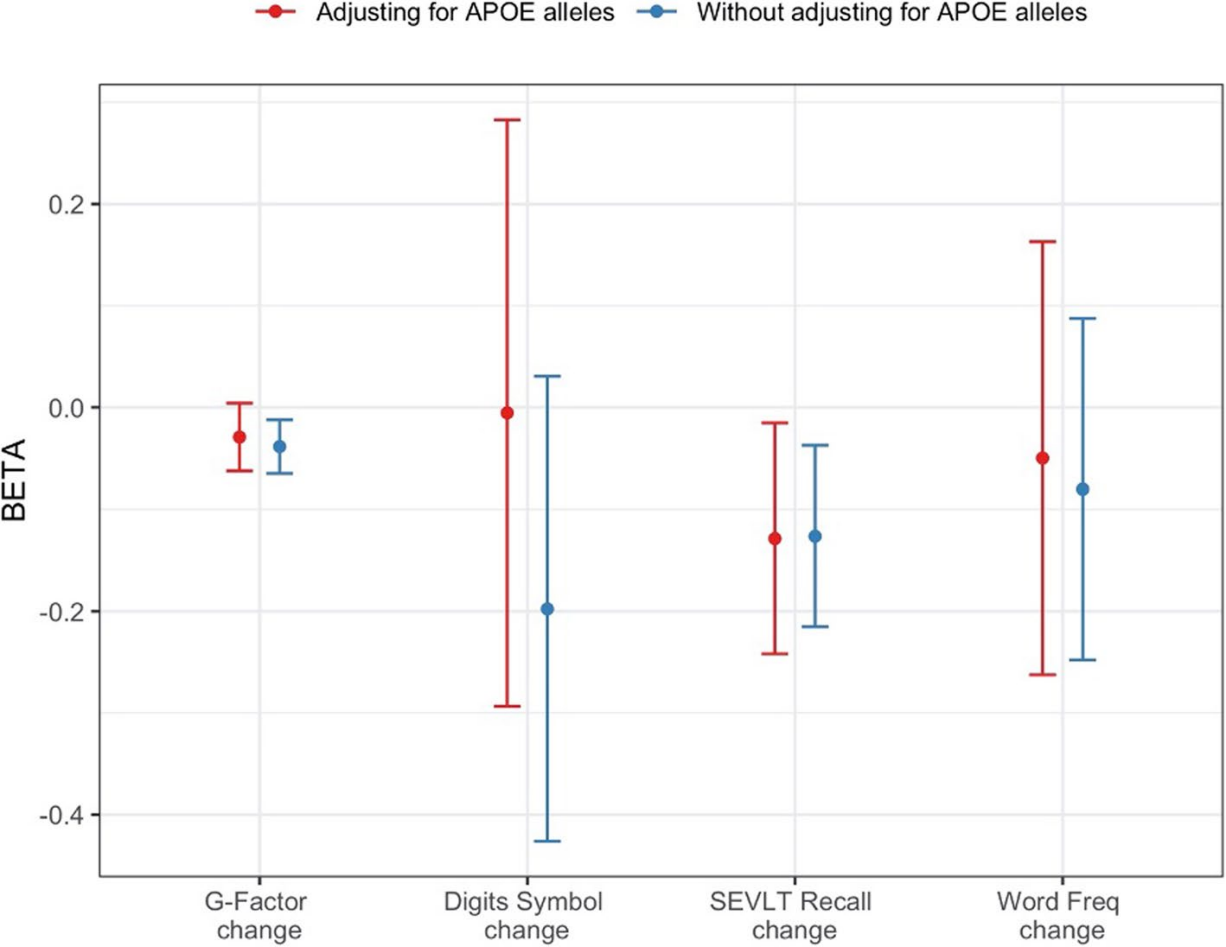
Association of the AD PRSsum with changes in cognitive functions. PRSsum was constructed as the unweighted sum of 5 standardized PRSs, each based on a separate GWAS described in Table 1. For each GWAS, the PRS were selected based on optimizing the coefficient of variation across 4 independent subsets of the SOL-INCA dataset. The figure provides the estimated effect size (beta) and 95% confidence intervals in the association of AD PRSsum with change in cognitive function from the baseline HCHS/SOL visit to the SOL-INCA exam. We provide results from three cognitive tests (digit symbol substitution, Brief Spanish English verbal learning tests (SEVLT), and word frequency, as well as from a change in a global measure of cognitive function, G-factor, computed based on the same tests. These phenotypes are described in the Supplementary Information. Effect estimates were obtained from linear mixed model implemented in the GENESIS R package, accounting for genetic relatedness, household, and block unit sharing via random effects, and adjusted for age at the HCHS/SOL baseline exam, time between exams, sex, study center, education level, *APOE* allele counts, and the first 5 principal components of genetic data as fixed effects. The sample sizes used for analyses in this figure ranged from *n* = 4,037 to *n* = 4,613. See Supplementary Table 5 for accurate numbers for each analysis

Figure 3 visualizes the association of PRSsum with changes in cognitive function between the baseline HCHS/SOL visit and the SOL-INCA examination, from linear mixed models adjusting for the same variables in the primary analysis described before for MCI (i.e. adjusted for education, as well as other standard variables, and with and without adjustment of *APOE* alleles). PRSsum was associated with reduced global cognition measured via the “G-factor”, as well as a reduction in performance in the B-SEVLT (Brief Spanish English verbal learning tests) recall test over time. PRSsum Associations were stronger in analyses that did not adjust for *APOE* alleles. Supplementary Table 7 provides the complete results, including comparison of PRSsum associations with the association of GWAS-specific PRSs.

## Discussion

We studied the association between PRSs for AD and MCI in the SOL-INCA study of diverse U.S. Hispanic/Latino adults. We constructed PRSs based on five GWAS, of individuals of European, African, Amerindian, and multi-ethnic heritage, and combined them in a simple sum, PRSsum, which formed the primary PRS. PRSsum was associated with MCI, as well as with change in global cognitive function. Surprisingly, PRSsum was associated with MCI while the *APOE*-$\epsilon 4$ allele alone was not. However, when removing *APOE*-region SNPs from the individual PRSs, and consequently, from PRSsum, the association with MCI weakened, reinforcing the *APOE* region contribution to the associations of AD PRSs with MCI.

We used the MGB Biobank dataset to validate our PRSs. In MGB Biobank the association of the PRSs are almost only due to AD and dementia cases, and almost entirely due to *APOE* SNPs. Thus, the MGB analysis confirms that the developed AD PRSs are indeed associated with AD and the strategy that generates PRSsum is useful, as PRSsum had the strongest association with MCI (including AD cases) compared to individual GWAS PRSs. Thus, the findings from this analysis suggest the MCI in HCHS/SOL may indeed capture a cognitive state that precedes AD, yet, we cannot rule out distinct genetic basis of MCI from AD. MCI-specific GWAS are needed to assess this distinction. As *APOE* alleles are not associated with MCI in SOL-INCA, while the AD PRS association is driven by *APOE* region SNPs, it is likely that different haplotypes or genetic patterns in the *APOE* regions are important in admixed Hispanic/Latino individuals.

A few other studies specifically looked at the association of AD PRS with cognitive decline and MCI in middle-aged individuals, i.e. in similar age groups to the SOL-INCA cohort. Logue et al. [31] considered AD PRS to predict MCI in non-Hispanic European ancestry individuals, and reported similar associations to those we observed: OR values between 1.17 to 1.4 (considering multiple p-value thresholds for including SNPs in the PRS) comparing cognitively normal adults and individuals with amnesic MCI. They also studied non-amnestic MCI, for which the association was weaker, suggesting heterogeneity of AD-related genetic association by type of MCI, or, in other words, heterogeneity in the underlying mechanisms of different types of MCI. The PRS constructed by Logue et al. [31], as well as by others, as reviewed in the introduction, were based on an earlier IGAP GWAS [16], from 2013, while we used an IGAP GWAS from 2019 [17], in addition to a few other GWASs. Other manuscripts developed a risk prediction model for cognitive decline using an IGAP GWAS-based PRS [45], and studied the association of an IGAP GWAS-based PRS with decline in multiple cognitive domains [46] (in non-Hispanic White individuals). In our dataset, PRSsum had a stronger association with MCI compared to individual GWAS PRSs. It is important to continue exploring the use of PRSsum and other PRS combination methods to leverage the increasing availability of published GWAS, especially in diverse populations.

An important question is whether our findings explain, in part, disparities in AD and MCI in Hispanics/Latinos, compared to European ancestry individuals and within Hispanic/Latino individuals of diverse backgrounds. While we still cannot answer this question, important observations are that PRSsum associations were fairly similar across subgroups defined by Hispanic/Latino background and by genetic ancestry. Moreover, *APOE*-$\epsilon 4$ allele count by itself was not associated with MCI but *APOE* region SNPs contributed to the PRS effectiveness. This can relate to either limited generalizability of findings from individuals of European ancestry to Hispanic/Latino individuals, distinct genetic basis of AD from MCI, or both. Either way, these findings also suggest the usefulness of using results from GWAS in diverse populations, as this analysis utilized results from multi-ancestry GWAS as well as GWAS in a population of African descent. A recent paper reported that PRS performance are reduced as the genetic distance between the training population (e.g., the population in which the GWAS was performed) and the testing population increases [47]. It will be interesting to use this approach with ancestry-specific PRSs over Hispanic/Latino groups. For now, our sample size is limited in achieving the required precision (evident by the overlapping confidence intervals when estimated PRS associations across background groups). Disparities in AD in Hispanics/Latinos are likely, at least in part, due to disparities in environmental and sociological exposures, such as air pollution [48], or socioeconomic status [49], which is also potentially associated with many environmental and psychological factors. These disparities may be associated with differences in genetic risk, and in gene-environment interactions, where environmental exposures exacerbate genetic risk. In future research we will use PRS developed here to study how environmental exposures modify PRS effects on MCI and AD.

Specific strengths of our study are the use of well phenotyped, yet understudied, diverse Hispanic/Latino cohort, comprehensive genetic data including proportions of global ancestries, and the use of multiple GWASs to construct PRS. Our study also has a few limitations. First, the MCI trait was not based on biological biomarkers, but rather on the NIA-AA criteria. Among people with cognitive performance from 1 to 2 SD below the mean of their peers (one of the criteria for defining MCI), some individual may have life-long below-average cognitive performance and are not on a trajectory of cognitive decline. However, we also required, according to the NIA-AA criteria, significant cognitive decline between the baseline and the SOL-INCA exam. Thus, individuals with life-long below-average cognitive performance are unlikely to be a substantial component of the MCI group. In longitudinal studies, many individuals who meet a clinical case definition for MCI one year revert to normal cognitive function in the next [50]. In addition, not all MCI is attributable to Alzheimer’s disease. Some individuals may have MCI attributable to vascular disease or other pathologic substrates. If the MCI case group includes many individuals who do not have MCI attributable to Alzheimer’s disease or mixed vascular and AD pathologies, that could lead to underestimation of the relative odds of MCI given a PRS and attenuate our ability to optimally identify PRS. Second, cognitive trajectories were estimated based on two points in time using cognitive tests with modest retest reliabilities. The low association of PRSs with indices of cognitive decline may reflect unreliability in the estimated slopes. An additional wave of follow-up data may strengthen our estimates of slope and our ability to identify PRS linked to cognitive trajectory. Third, we did not have a similar population to train or validate the PRS. We used an internal validation approach, and then validated the PRS in MGB Biobank, a healthcare-based population. In future work we will build upon new datasets, e.g., from the All of Us cohort, to study AD PRS in a large and more diverse population of Hispanic/Latino adults. Finally, because no GWAS of AD is available in Hispanic/Latino populations, we were not able to use recently proposed methods designed to leverage information from, typically, European populations, to other populations, such as of Hispanic/Latino individuals [51, 52].

In summary, we used summary statistics from AD GWAS to construct multiple PRSs, and combined them as PRSsum, which was associated with MCI in U.S. Hispanics/Latinos. While most individual GWAS-based PRSs were also associated with MCI, only PRSsum was associated with cognitive decline. The *APOE*-$\epsilon 4$ allele was not associated with MCI in SOL-INCA, but *APOE* region SNPs substantially contributed to the association of AD PRS with MCI. This findings adds to the growing literature suggesting ancestry-specific genetic components in the *APOE* region associated with cognitive aging outcomes in non-White populations [53–56]. Cognitive aging may be the result of other health and disease phenotypes, such as diabetes and poor kidney function, and sleep disturbances [57]. In future work we will study genetic prediction of cognitive decline in Hispanic/Latino adults using PRS for risk factors for cognitive aging, in addition to AD PRS, while accounting for lifestyle and other risk factors.

### Supplementary Information

**Additional file 1. **

## Data Availability

HCHS/SOL genetic and phenotypic data can be obtained through the study's Data Coordinating Center using an approved data use agreement. Information is provided in https://sites.cscc.unc.edu/hchs/. HCHS/SOL genetic and phenotypic data can also be obtained from dbGaP under accession number phs000810.v1.p1. GWAS summary statistics used to develop PRSs are available as described in Table 1. Instructions to construct the developed PRSs, in the form of list of variant, alleles, and weights, as well as example PRSice command to generate them from plink files and R code to combine them into PRSsum will be available on GitHub as well as on the PGS Catalog upon paper acceptance.
