# Supplementary material for: A polygenic risk score for Alzheimer’s disease constructed using APOE-region variants has stronger association than APOE alleles with mild cognitive impairment in Hispanic/Latino adults in the U.S
Source: Alzheimers Res Ther. 2023 Aug 30;15:146. doi: 10.1186/s13195-023-01298-3 (PMC10469805; doi:10.1186/s13195-023-01298-3)
Supplement: Supplementary file 1 — Additional file 1. [file 13195_2023_1298_MOESM1_ESM.docx]

Supplementary Information to:

A polygenic risk score for Alzheimer’s Disease constructed using APOE-region variants has stronger association than APOE alleles with Mild Cognitive Impairment in Hispanic/Latino adults in the U.S.

Sofer, Kurniansyah et al.

*Correspondence: Tamar Sofer, tsofer@bidmc.harvard.edu

[Supplementary methods 2](#_Toc141888574)

[The Hispanic Community Health Study/Study of Latinos 2](#_Toc141888575)

[Cognitive phenotypes 3](#_Toc141888576)

[Cognitive phenotypes used in secondary analyses 4](#_Toc141888577)

[Genotyping and imputation in HCHS/SOL 4](#_Toc141888578)

[MGB Biobank methods 5](#_Toc141888579)

[Supplementary figures 7](#_Toc141888580)

[Supplementary Figure 1. Estimated effect sizes and confidence intervals of FinnGen-based PRS in association with MCI 7](#_Toc141888581)

[Supplementary Figure 2. Estimated effect sizes and confidence intervals of Jun et al.-based PRS in association with MCI 8](#_Toc141888582)

[Supplementary Figure 3. Estimated effect sizes and confidence intervals of Kunkle et al. 2019-based PRS in association with MCI 9](#_Toc141888583)

[Supplementary Figure 4. Estimated effect sizes and confidence intervals of Kunkle et al. 2021-based PRS in association with MCI 10](#_Toc141888584)

[Supplementary Figure 5. Estimated effect sizes and confidence intervals of Bellenguez et al.-based PRS in association with MCI 11](#_Toc141888585)

[Supplementary Figure 6. Estimated effect sizes and confidence intervals of Bellenguez et al. lead variants -based PRS in association with MCI 12](#_Toc141888586)

[Supplementary Figure 7. Estimated effect sizes and confidence intervals of Lake et al. -based PRS in association with MCI 13](#_Toc141888587)

[Supplementary Figure 8: Distribution of PRSs stratified by *APOE*-$\epsilon4$ carriers and non-carriers. 14](#_Toc141888588)

[Supplementary Figure 9: Distribution of PRSs stratified by *APOE*-$\epsilon4$ carriers and non-carriers for European ancestry(>=80 %) 15](#_Toc141888589)

[Supplementary Tables 16](#_Toc141888590)

[Supplementary Table 1: Characteristics of the AD PRS selected in association with MCI in the SOL-INCA dataset 16](#_Toc141888591)

[Supplementary Table 2: Coefficients of variation of the AD PRSs constructed using Bayesian methods in association with MCI in the SOL-INCA dataset 16](#_Toc141888592)

[Supplementary Table 3: Test of differences in R^2^ prediction performance between PRSsum and each of its component PRSs. 17](#_Toc141888593)

[Supplementary Table 4. Estimated association of AD PRS and of APOE allele counts with MCI excluding MCI+ participants 18](#_Toc141888594)

[Supplementary Table 5: Characteristics of the MGB Biobank dataset participants 19](#_Toc141888595)

[Supplementary Table 6: Associations of PRS selected in the SOL-INCA dataset with MCI in the MGB Biobank dataset 20](#_Toc141888596)

[Supplementary Table 7: Associations of selected PRS with changes in cognitive functions in SOL-INCA 21](#_Toc141888597)

[References 23](#_Toc141888598)

# Supplementary methods

## The Hispanic Community Health Study/Study of Latinos

The HCHS/SOL is a population-based longitudinal cohort study established to study risk and protective factors in cardiovascular disease development among U.S. Hispanics/Latinos [1]. The study follows Hispanic/Latino participants from four metropolitan areas: Bronx NY, Miami FL, Chicago IL, and San Diego CA, with 16,415 participants aged 18-74 years examined in the baseline visit. Individuals were recruited via a probability sampling scheme from pre-defined census block units, chosen to provide diversity with respect to socioeconomic status as well as national origin or background [2]. Participants self-identified with six Hispanic/Latino background groups: Central American, South American, Mexican (Mainland groups, have high Amerindian genetic ancestry and low African ancestry), Cuban, Dominican, and Puerto-Rican (Caribbean group, have low Amerindian ancestry, and high African ancestry). At baseline, participants who were at least 45 years old and did not refuse nor had health limitations (n=9,714) were administered cognitive tests, including a Brief Spanish English verbal learning tests (B-SEVLT; [3]), assessing episodic learning and memory; word frequency test (also called word fluency, WF; [4]), assessing verbal function; and digit symbol substitution test (DSS; [5]), assessing processing speed and executive function. A second clinic visit occurred in 2014-2017, and during or after this visit, 6,377 participants who were eligible (completed neurocognitive testing during visit 1 and were at least 50 years old at visit 2) participated in the Study of Latinos-Investigation of Neurocognitive Aging (SOL-INCA), an ancillary study to the HCHS/SOL. SOL-INCA exams occurred, on average, 7 years after the baseline visit. Detailed information about the SOL-INCA exam and cognitive phenotyping is available in and [6]. In brief, the same cognitive battery from the baseline visit was applied and complemented with additional neurocognitive tests and instrumental activities of daily living questionnaire to assess functional status. In this study, we included n=4,256 individuals who participated in both the SOL-INCA study and were genotyped (as detailed below). All individuals provided written informed consent at their recruitment site.

## Cognitive phenotypes

Our primary phenotype was MCI, defined according to the National Institute on Aging-Alzheimer’s Association (NIA-AA) criteria [7], as detailed in [8]. In brief, a participant was classified with MCI if the three conditions were satisfied: (a) any cognitive test score in the range -1 to -2 SD (standard deviation) of the SOL-INCA robust norms adjusted for age, sex, education, and Picture Vocabulary Test scores, (b) a global measure of cognition declined by more than 0.055 SD per year between the visits, and (c) participant self-reported cognitive decline based on an Everyday Cognition questionnaire. Finally, we also included in the MCI group 62 individuals with MCI+ (suspect severe impairment), defined by a “cognitive deficit” of any cognitive test score lower than -2 SD compared to the internal norms, and more than minimal functional impairment according to the instrumental daily living questionnaire.

## Cognitive phenotypes used in secondary analyses

In secondary analysis, we also report PRS associations with changes in specific test scores measured in both the baseline and the SOL-INCA visits: B-SEVLT, WF, and DSS, and with a cognitive decline variable computed as a change in G-factor. To compute this variable, we used the baseline cognitive tests (B-SEVLT recall, DSS, and WF) restricted to the set of people who participated in the SOL-INCA exam. We computed the mean and standard deviation (SD) of each of the tests during the baseline visit while accounting for study design and sampling into the SOL-INCA study. We used these estimated means and SDs to scale the cognitive test values in each of the baseline and SOL-INCA exams (we subtracted the scores of each test by the corresponding mean and divided by the corresponding SD). We then computed principal components based on the standardized baseline exam test scores and obtained loadings for each of the three (standardized) tests. We used the loadings to compute the first principal component scores in both the baseline and the SOL-INCA visit to obtain G_baseline_ and G_inca_. The change in global cognitive function was computed as G_change_ = G_inca_ - G_baseline_. Negative values indicate decline in global cognitive function between the baseline and the SOL-INCA exams. Note that some values were positive, but the trend across all participants was of decline.

## Genotyping and imputation in HCHS/SOL

Blood was drawn from HCHS/SOL participants during the baseline exam. Individuals who consented to genetic studies were genotyped using an Illumina Omni2.5M array, which included 150,000 custom-selected Single Nucleotide Polymorphisms (SNPs), including ancestry-informative and Amerindian-specific variants. Global ancestry proportions measuring the proportion of the genome inherited from European, African, and Amerindian ancestors, and genetic principal components, were computed as previously reported [9]. The genotypes were imputed to the Trans-Omics in Precision Medicine (TOPMed) freeze 5b reference panel as described in [10]. APOE genotyping was performed separately using a TaqMan assay as previously described [11].

## MGB Biobank methods

Samples, genomic data, and health information were obtained from the Mass General Brigham (MGB) Biobank, a biorepository of consented patient samples at Mass General Brigham.

**DNA samples**

DNA samples are processed from whole blood that was collected as a dedicated research draw or as a clinical discard. Dedicated research samples are aimed to be processed within four hours of collection. Clinical discards are processed 24+ hours after collection. Whole blood is spun to buffy coat with a centrifuge and the buffy coat is stored in a freezer up to several months. The buffy coat is then extracted to DNA. The DNA is then placed in an ultralow freezer (-80ºC).Each DNA aliquot contains a minimum of 2 ug of DNA. The concentration varies.

**Genotyping**

Samples have been genotyped using three versions of the biobank SNP array offered by Illumina that is designed to capture the diversity of genetic backgrounds across the globe. The first batch of data was generated on the Multi-Ethnic Genotyping Array (MEGA) array, the first release of this SNP array. The second, third, and fourth batches were generated on the Expanded Multi-Ethnic Genotyping Array (MEGA Ex) array. All remaining data were generated on the Multi-Ethnic Global (MEG) BeadChip.

**Imputation**

Prior to performing imputation, files were converted to VCF format, separated by chromosomes. When multiple probes measured the same genotypes, they were checked for concordance and were set to a missing value if the genotypes did not match. Files were uploaded to the Michigan Imputation Server, and Genotypes were imputed using TOPMed reference panel. Genomic coordinates are provided in GRCh38.

**Quality control**

We performed quality control using PLINK (v2.0. We filtered SNPs with low-quality imputation (r < 0.5), with missing call rates > 0.1, HWE p-value less than 1x10^-6^ and MAF <1%.

We computed principal component (PC) using PLINK: we pruned the genotype data using a window size of 1000 variants, sliding across the genome with a step size of 250 variants at a time, filtering out any SNPs with LD R^2^>0.1. We used unrelated individuals (3rd degree, identified using PLINK) to compute the loadings for the first 10 PCs.

# Supplementary figures

## Supplementary Figure 1. Estimated effect sizes and confidence intervals of FinnGen-based PRS in association with MCI


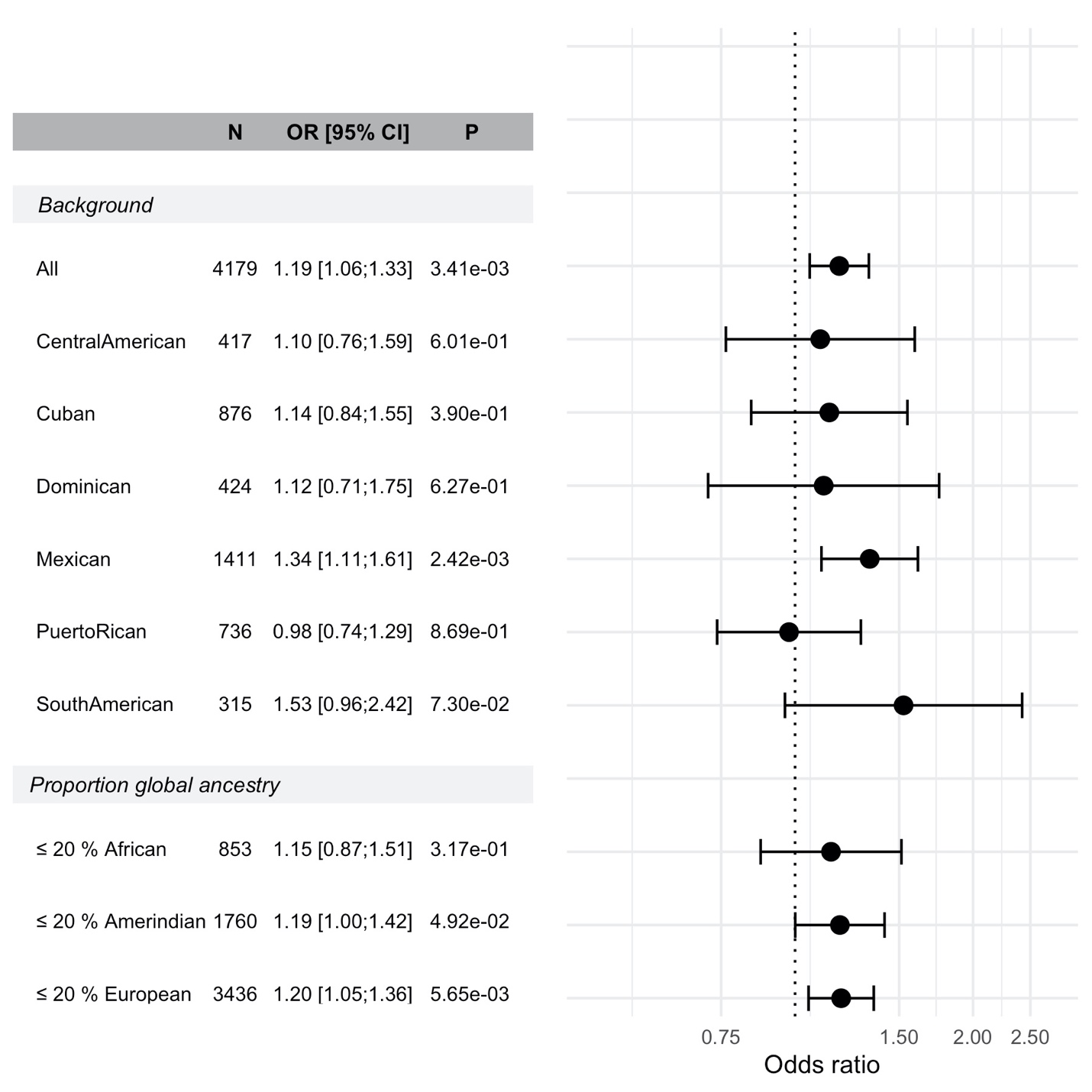


PRS method and tuning parameters were selected based on optimizing the coefficient of variation across 4 independent subsets of the SOL-INCA dataset. We provide the effect size, confidence interval, and p-value in models based on the complete dataset (“All”), by Hispanics/Latino background, and for the subsets of people with at least 20% global proportion of African, Amerindian, and European ancestries. The PRS association was estimated in a model adjusted for age at the HCHS/SOL baseline visit, time from HCHS/SOL baseline to the SOL-INCA visit, sex, study center, 5 principal components, and APOE-$\epsilon4$ and APOE-$\epsilon2$ allele counts.

## Supplementary Figure 2. Estimated effect sizes and confidence intervals of Jun et al.-based PRS in association with MCI


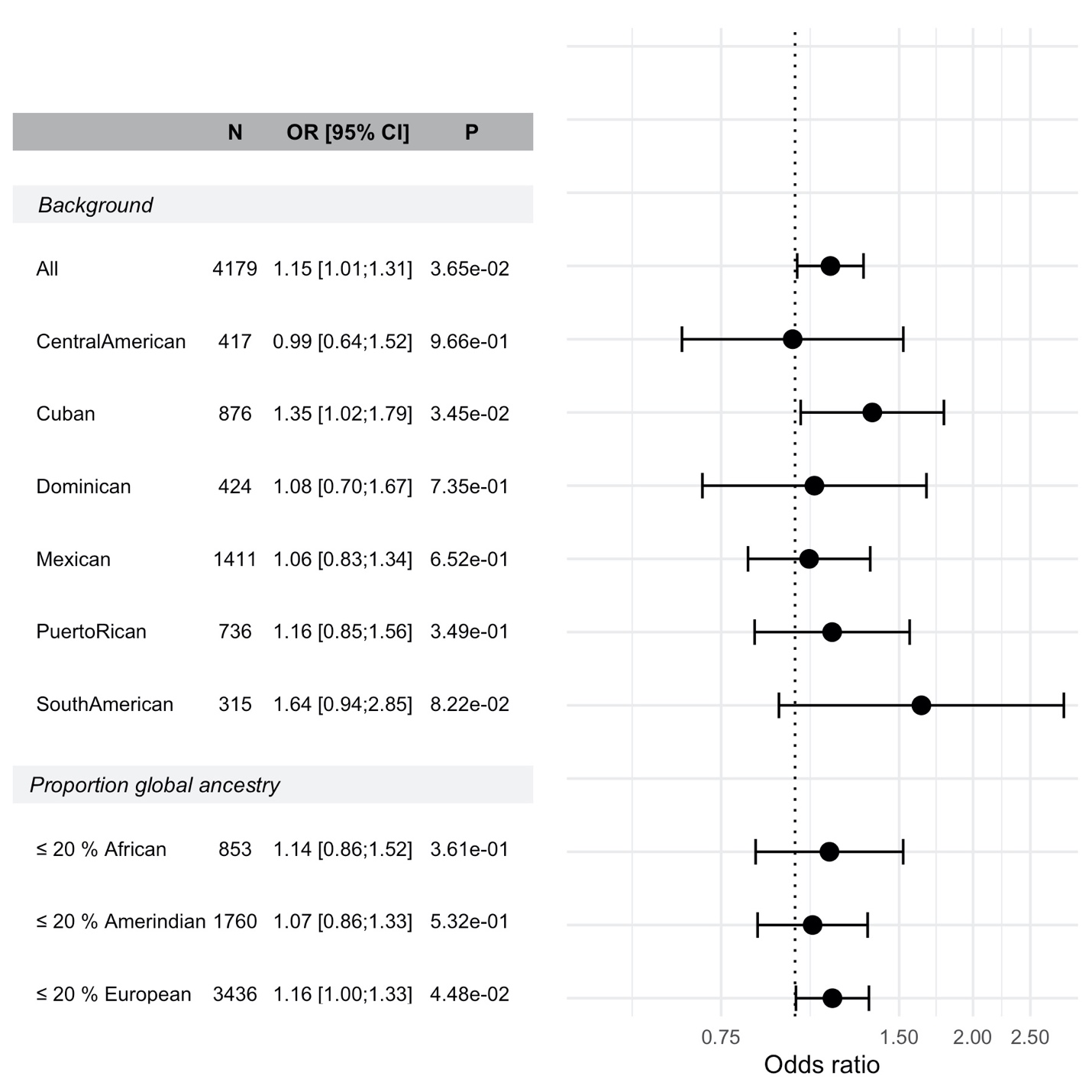


PRS method and tuning parameters were selected based on optimizing the coefficient of variation across 4 independent subsets of the SOL-INCA dataset. We provide the effect size, confidence interval, and p-value in models based on the complete dataset (“All”), by Hispanics/Latino background, and for the subsets of people with at least 20% global proportion of African, Amerindian, and European ancestries. The PRS association was estimated in a model adjusted for age at the HCHS/SOL baseline visit, time from HCHS/SOL baseline to the SOL-INCA visit, sex, study center, 5 principal components, and APOE-$\epsilon4$ and APOE-$\epsilon2$ allele counts.

## Supplementary Figure 3. Estimated effect sizes and confidence intervals of Kunkle et al. 2019-based PRS in association with MCI


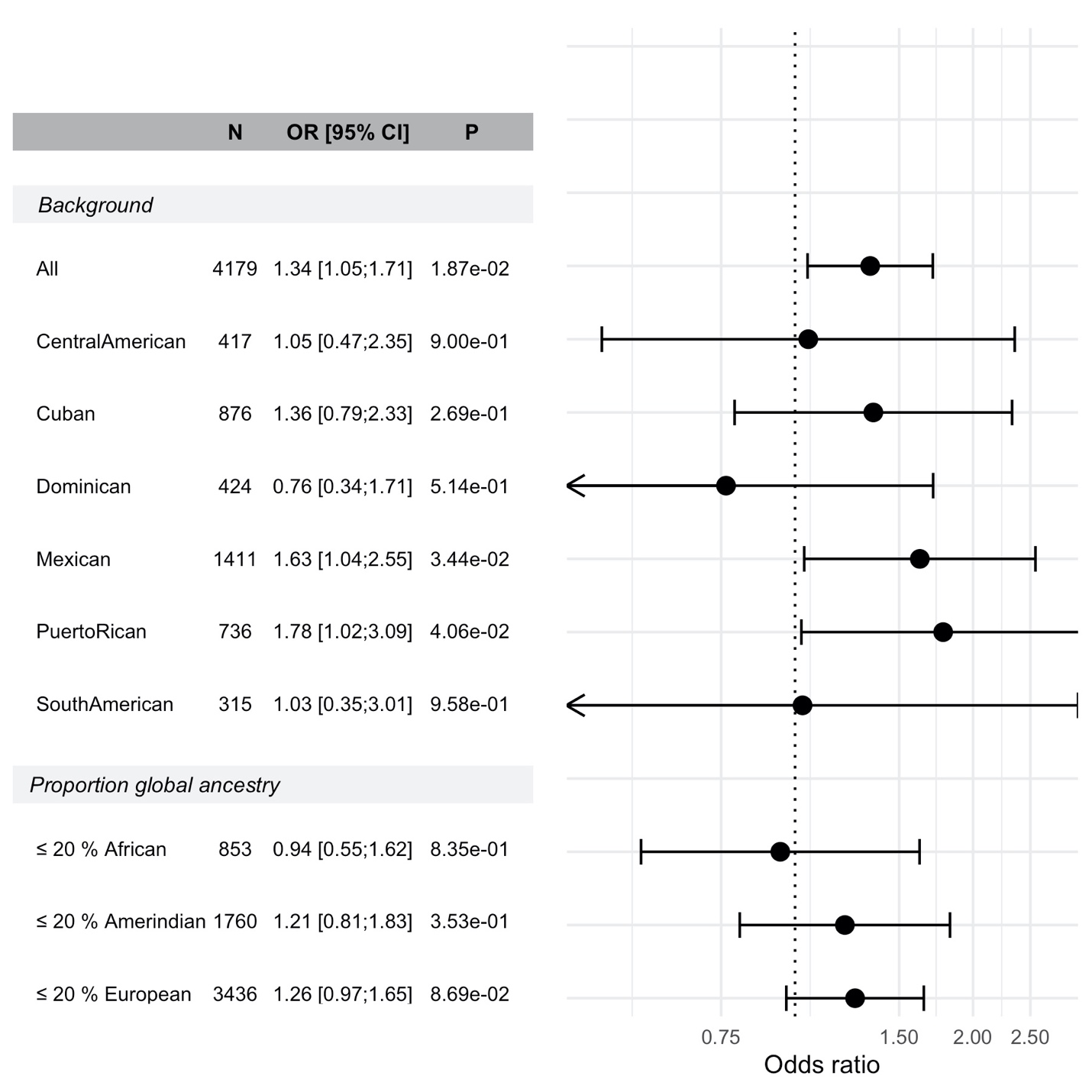


PRS method and tuning parameters were selected based on optimizing the coefficient of variation across 4 independent subsets of the SOL-INCA dataset. We provide the effect size, confidence interval, and p-value in models based on the complete dataset (“All”), by Hispanics/Latino background, and for the subsets of people with at least 20% global proportion of African, Amerindian, and European ancestries. The PRS association was estimated in a model adjusted for age at the HCHS/SOL baseline visit, time from HCHS/SOL baseline to the SOL-INCA visit, sex, study center, 5 principal components, and APOE-$\epsilon4$ and APOE-$\epsilon2$ allele counts.

## Supplementary Figure 4. Estimated effect sizes and confidence intervals of Kunkle et al. 2021-based PRS in association with MCI


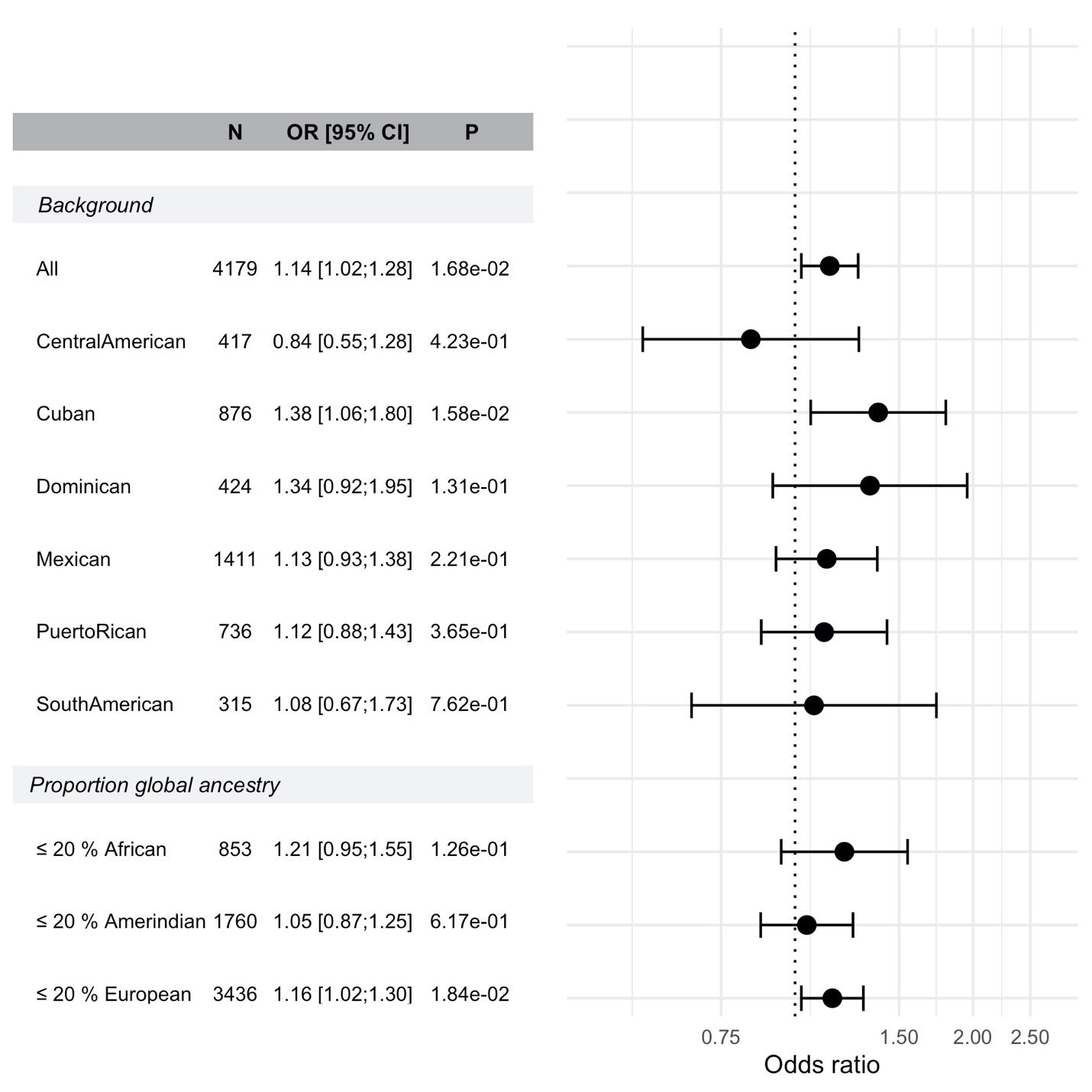


PRS method and tuning parameters were selected based on optimizing the coefficient of variation across 4 independent subsets of the SOL-INCA dataset. We provide the effect size, confidence interval, and p-value in models based on the complete dataset (“All”), by Hispanics/Latino background, and for the subsets of people with at least 20% global proportion of African, Amerindian, and European ancestries. The PRS association was estimated in a model adjusted for age at the HCHS/SOL baseline visit, time from HCHS/SOL baseline to the SOL-INCA visit, sex, study center, 5 principal components, and APOE-$\epsilon4$ and APOE-$\epsilon2$ allele counts.

## Supplementary Figure 5. Estimated effect sizes and confidence intervals of Bellenguez et al.-based PRS in association with MCI


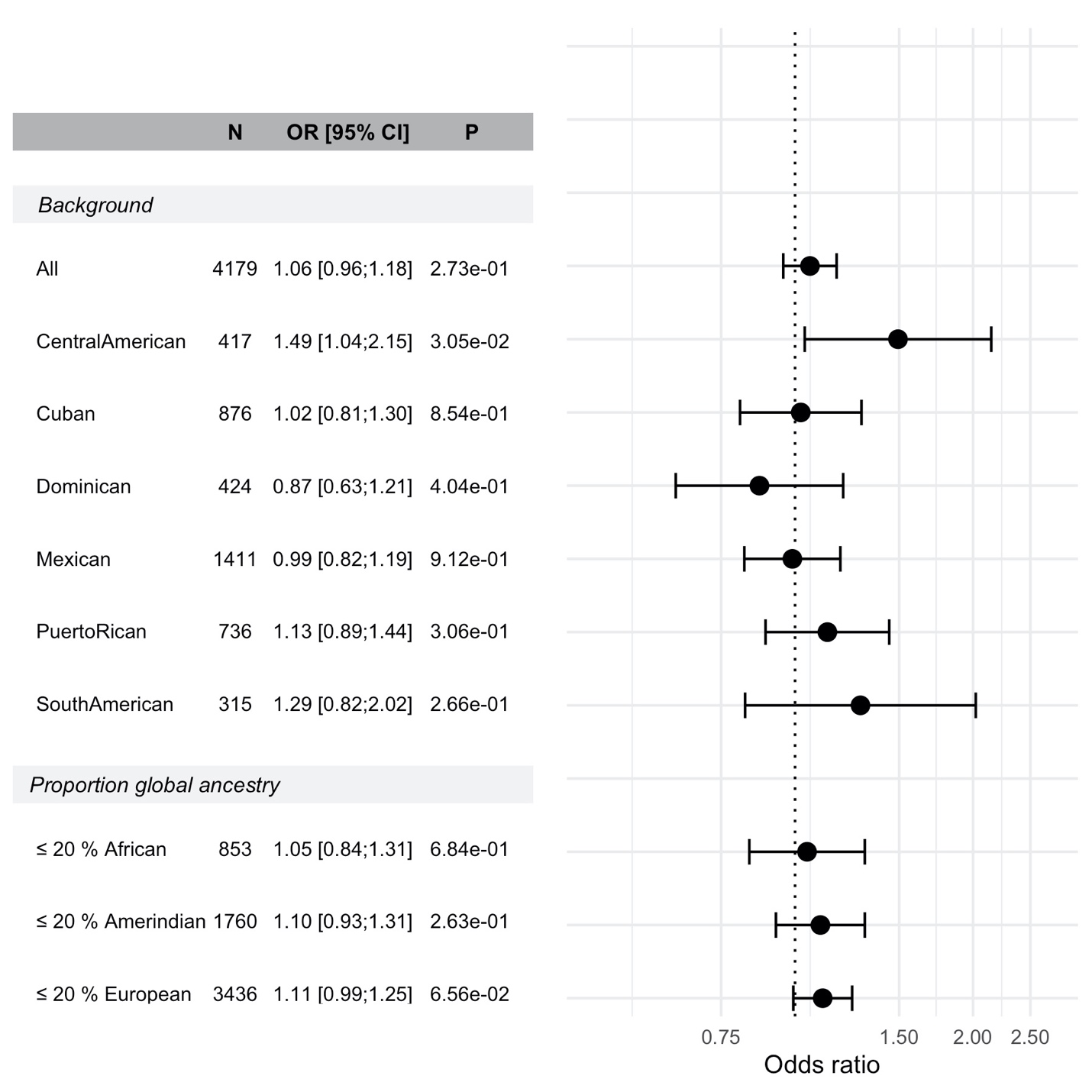


PRS method and tuning parameters were selected based on optimizing the coefficient of variation across 4 independent subsets of the SOL-INCA dataset. We provide the effect size, confidence interval, and p-value in models based on the complete dataset (“All”), by Hispanics/Latino background, and for the subsets of people with at least 20% global proportion of African, Amerindian, and European ancestries. The PRS association was estimated in a model adjusted for age at the HCHS/SOL baseline visit, time from HCHS/SOL baseline to the SOL-INCA visit, sex, study center, 5 principal components, and APOE-$\epsilon4$ and APOE-$\epsilon2$ allele counts.

## Supplementary Figure 6. Estimated effect sizes and confidence intervals of Bellenguez et al. lead variants -based PRS in association with MCI

**
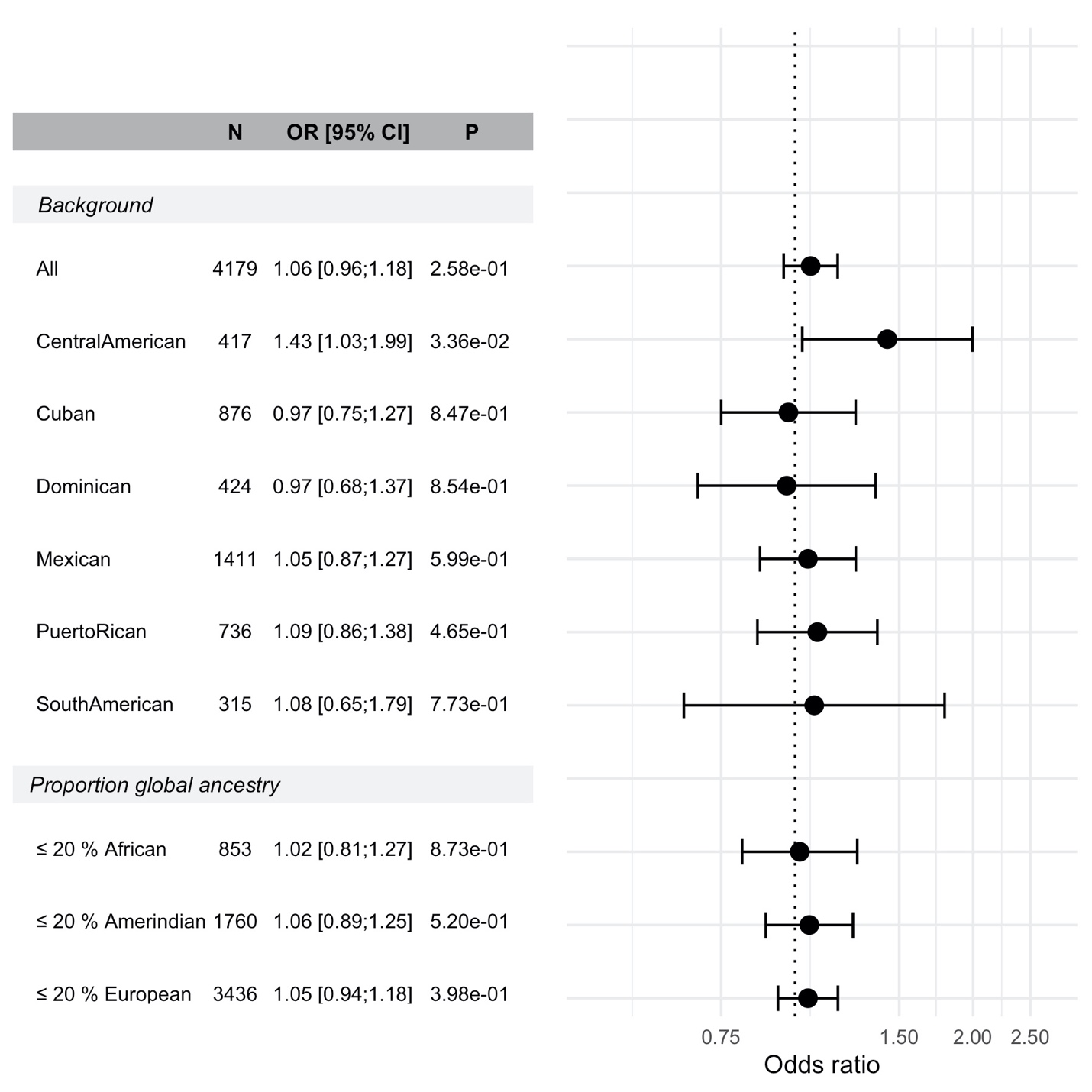
**

The PRS used lead variants with weights being the effect estimates (log ORs) reported in Bellenguez et al. We provide the effect size, confidence interval, and p-value in models based on the complete dataset (“All”), by Hispanics/Latino background, and for the subsets of people with at least 20% global proportion of African, Amerindian, and European ancestries. The PRS association was estimated in a model adjusted for age at the HCHS/SOL baseline visit, time from HCHS/SOL baseline to the SOL-INCA visit, sex, study center, 5 principal components, and APOE-$\epsilon4$ and APOE-$\epsilon2$ allele counts.

## Supplementary Figure 7. Estimated effect sizes and confidence intervals of Lake et al. -based PRS in association with MCI


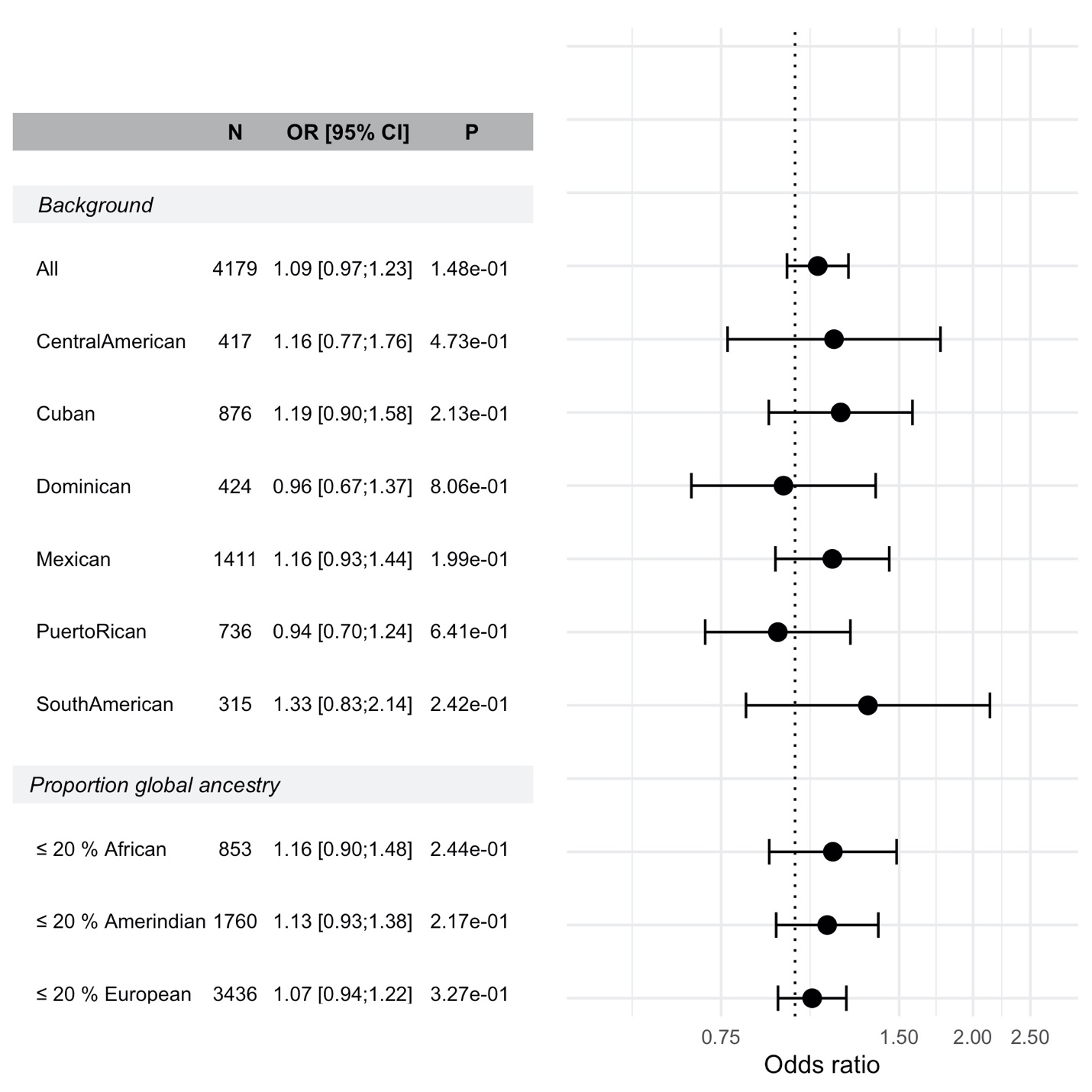


PRS method and tuning parameters were selected based on optimizing the coefficient of variation across 4 independent subsets of the SOL-INCA dataset. We provide the effect size, confidence interval, and p-value in models based on the complete dataset (“All”), by Hispanics/Latino background, and for the subsets of people with at least 20% global proportion of African, Amerindian, and European ancestries. The PRS association was estimated in a model adjusted for age at the HCHS/SOL baseline visit, time from HCHS/SOL baseline to the SOL-INCA visit, sex, study center, 5 principal components, and APOE-$\epsilon4$ and APOE-$\epsilon2$ allele counts.

## Supplementary Figure 8: Distribution of PRSs stratified by *APOE*-$\epsilon4$ carriers and non-carriers.

**
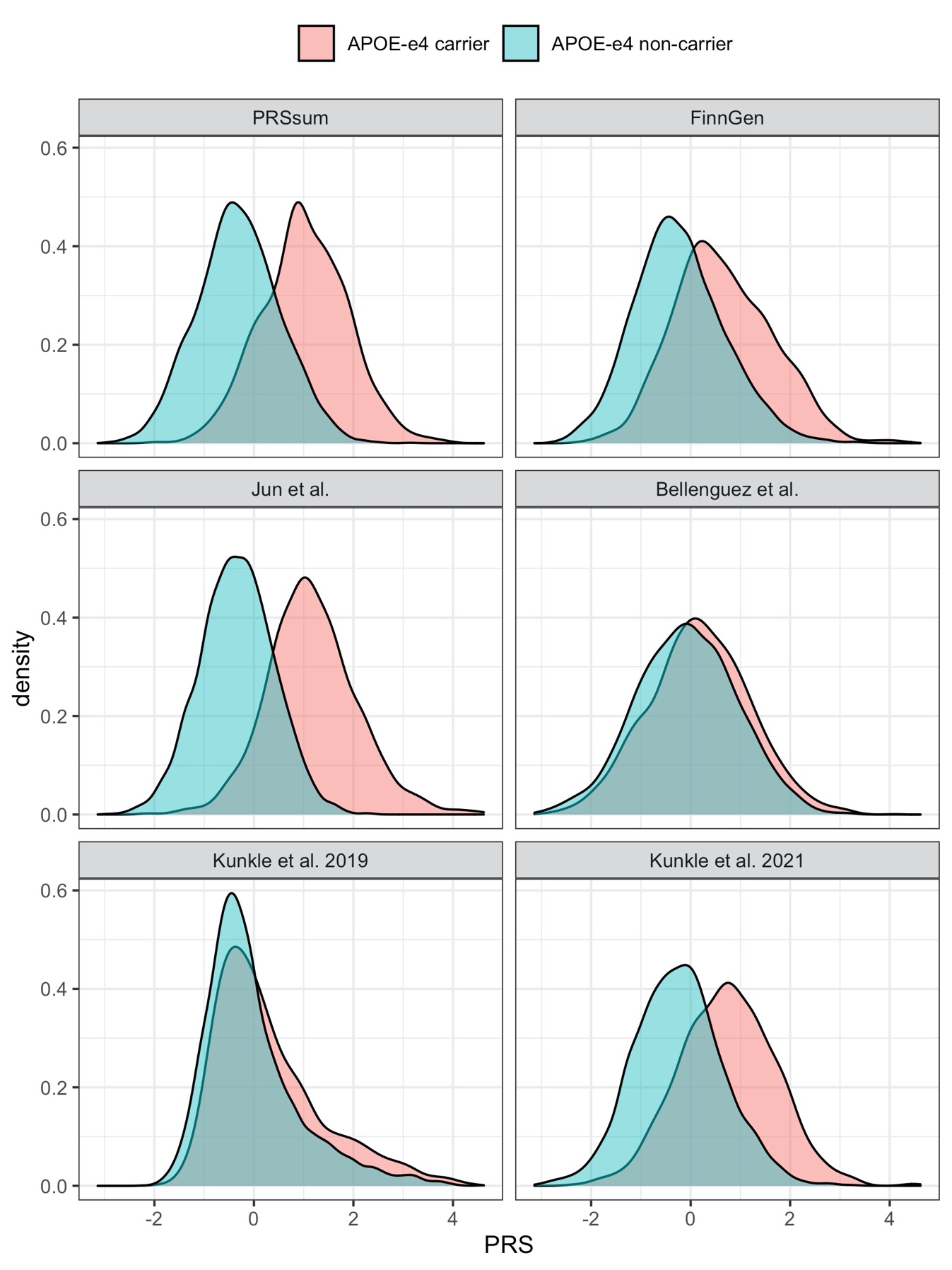
**

## Supplementary Figure 9: Distribution of PRSs stratified by *APOE*-$\epsilon4$ carriers and non-carriers for European ancestry(>=80 %)


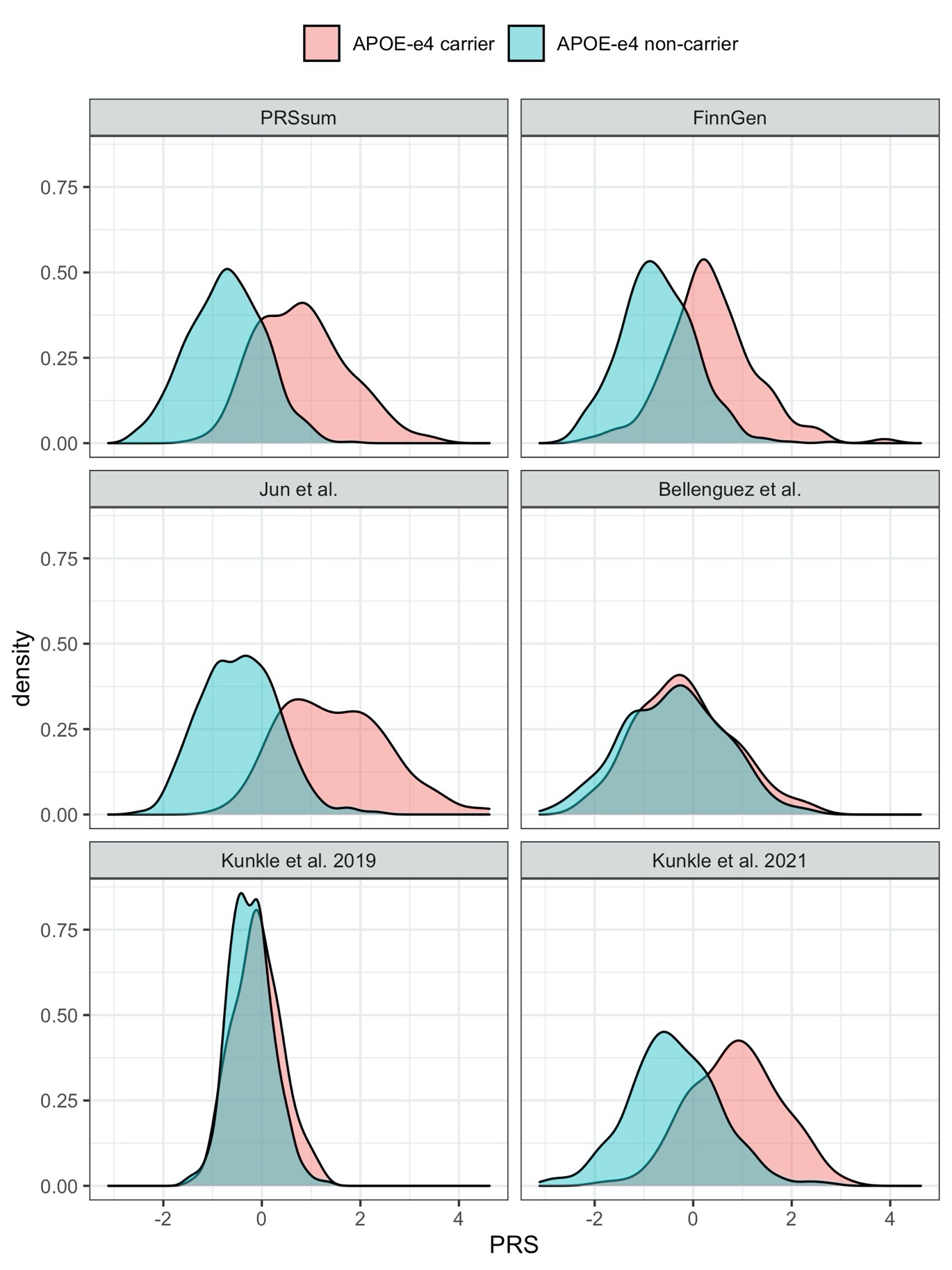


# Supplementary Tables

## Supplementary Table 1: Characteristics of the AD PRS selected in association with MCI in the SOL-INCA dataset

| Study | GWAS Population | # SNP | R^2^ | Distance | Threshold | CV |
| --- | --- | --- | --- | --- | --- | --- |
| Bellenguez et al. | White | 1937 | 0.1 | 1000kb | 1.00E-03 | 0.70 |
| FinnGen | Finish | 81 | 0.1 | 250kb | 1.00E-06 | 1.03 |
| Jun et al. | Multi-Ethnic | 85 | 0.2 | 250kb | 1.00E-05 | 0.82 |
| Kunkle et al. 2019 | White | 12002 | 0.3 | 1000kb | 1.00E-02 | 0.83 |
| Kunkle et al. 2021 | Black | 157 | 0.3 | 250kb | 1.00E-04 | 1.10 |
| Lake et al. 2023 | Multi-Ethnic | 53255 | 0.1 | 500kb | 1.00E-01 | 0.82 |

For each GWAS, the table describes the characteristics of the PRSice-based PRS selected by optimizing the coefficient of variation (CV) across 4 independent subsets of the data. R^2^ and “Distance” are parameters used for clumping. Threshold is the p-value threshold. PRSs developed by PRS-CS were considered but did not optimize the CV and were not selected.

## Supplementary Table 2: Coefficients of variation of the AD PRSs constructed using Bayesian methods in association with MCI in the SOL-INCA dataset

| Study | GWAS Population | PRS  Method | CV |
| --- | --- | --- | --- |
| Bellenguez et al. | White | PRS-CS | 28.67 |
| FinnGen | Finish | PRS-CS | 4.06 |
| Jun et al. | Multi-Ethnic | LDPred2 | 1.00 |
| Kunkle et al. 2019 | White | PRS-CS | 4.38 |
| Kunkle et al. 2021 | Black | PRS-CS | 70.44 |
| Lake et al. 2023 | Multi-Ethnic | LDpred2 | 4.13 |

## Supplementary Table 3: Test of differences in R^2^ prediction performance between PRSsum and each of its component PRSs.

| **Study** | **GWAS Population** | **R^2^** | **P-value based**  **on two-sided**  **hypothesis** | **P-value based**  **on one-sided**  **hypothesis** |
| --- | --- | --- | --- | --- |
| Bellenguez et al. | White | 0.0004 | 0.2286 | 0.1143 |
| FinnGen | Finish | 0.0012 | 0.6558 | 0.3279 |
| Jun et al. | Multi-Ethnic | 0.0007 | 0.2801 | 0.1400 |
| Kunkle et al. 2019 | White | 0.0003 | 0.2252 | 0.1126 |
| Kunkle et al. 2021 | Black | 0.0005 | 0.2829 | 0.1415 |

The estimated R^2^ for PRSsum was 0.0016.

## Supplementary Table 4. Estimated association of AD PRS and of APOE allele counts with MCI excluding MCI+ participants

| **Study** | **GWAS Population** | **#SNP** | ***APOE-***$\boldsymbol{\epsilon}$***2*** | **P** | ***APOE-***$\boldsymbol{\epsilon}$***4*** | **P** | **PRS** | **P** |
| --- | --- | --- | --- | --- | --- | --- | --- | --- |
| PRS includes *APOE* region SNPs, analysis adjusted for *APOE* alleles | | | | | | | | |
| FinnGen | Finish | 81 | 0.98[0.66;1.45] | 0.91 | 0.96[0.73;1.26] | 0.75 | 1.19[1.05;1.34] | 5.30E-03 |
| Jun et al. | Multi-Ethnic | 85 | 0.91[0.62;1.35] | 0.64 | 0.97[0.70;1.34] | 0.85 | 1.09[0.95;1.26] | 0.20 |
| Bellenguez et al. | White | 1937 | 0.92[0.62;1.37] | 0.68 | 1.11[0.86;1.43] | 0.43 | 1.02[0.92;1.14] | 0.69 |
| Kunkle et al. 2019 | White | 12002 | 0.93[0.63;1.38] | 0.74 | 1.08[0.84;1.40] | 0.53 | 1.28[0.98;1.66] | 0.07 |
| Kunkle et al. 2021 | Black | 157 | 0.91[0.61;1.34] | 0.62 | 0.98[0.74;1.29] | 0.87 | 1.14[1.01;1.28] | 0.03 |
| PRSsum | Multi-Ethnic | 14100 | 0.96[0.65;1.43] | 0.86 | 0.87[0.64;1.18] | 0.37 | 1.22[1.06;1.41] | 4.67E-03 |
| PRS excludes *APOE* region SNPs, analysis adjusted for *APOE* alleles | | | | | | | | |
| FinnGen | Finish | 34 | 0.92[0.62;1.36] | 0.69 | 1.10[0.85;1.41] | 0.48 | 1.17[1.05;1.31] | 5.57E-03 |
| Jun et al. | Multi-Ethnic | 38 | 0.91[0.62;1.35] | 0.65 | 1.11[0.86;1.43] | 0.41 | 1.00[0.90;1.12] | 0.95 |
| Bellenguez et al. | White | 1871 | 0.91[0.62;1.35] | 0.65 | 1.11[0.86;1.43] | 0.41 | 1.00[0.89;1.11] | 0.94 |
| Kunkle et al. 2019 | White | 11805 | 0.92[0.62;1.36] | 0.66 | 1.11[0.86;1.43] | 0.41 | 1.21[0.93;1.58] | 0.15 |
| Kunkle et al. 2021 | Black | 146 | 0.91[0.62;1.35] | 0.64 | 1.11[0.86;1.43] | 0.41 | 1.10[0.99;1.23] | 0.07 |
| PRSsum | Multi-Ethnic | 13807 | 0.92[0.62;1.36] | 0.67 | 1.11[0.86;1.43] | 0.42 | 1.14[1.01;1.28] | 0.03 |
| PRS includes *APOE* region SNPs, analysis unadjusted for *APOE* alleles | | | | | | | | |
| FinnGen | Finish | 81 | -- | -- | -- | -- | 1.17[1.05;1.30] | 3.37E-03 |
| Jun et al. | Multi-Ethnic | 85 | -- | -- | -- | -- | 1.07[0.96;1.18] | 0.21 |
| Bellenguez et al. | White | 1937 | -- | -- | -- | -- | 1.03[0.93;1.14] | 0.60 |
| Kunkle et al. 2019 | White | 12002 | -- | -- | -- | -- | 1.23[0.96;1.57] | 0.10 |
| Kunkle et al. 2021 | Black | 157 | -- | -- | -- | -- | 1.11[1.00;1.23] | 0.05 |
| PRSsum | Multi-Ethnic | 14100 | -- | -- | -- | -- | 1.16[1.04;1.29] | 9.74E-03 |

## Supplementary Table 5: Characteristics of the MGB Biobank dataset participants

|  | Overall |
| --- | --- |
| N | 23158 |
| Age (mean (SD)) | 68.00 (10.04) |
| Gender F (%) | 11921 (51.5) |
| Race/ethnicity (%) |  |
| Black | 1005 ( 4.3) |
| Asian | 306 ( 1.3) |
| White | 20348 (87.9) |
| Hispanic/Latino | 614 ( 2.7) |
| Other | 366 ( 1.6) |
| Unknown | 518 ( 2.2) |
| MCI Yes (%) | 885 ( 3.8) |
| AD/dementia Yes (%) | 320 (1.3) |

## Supplementary Table 6: Associations of PRS selected in the SOL-INCA dataset with MCI in the MGB Biobank dataset

| **Study** | **GWAS Population** | **R2** | **Distance** | **Threshold** | **OR** | **SE** | **P-value** | **OR** | **SE** | **P-value** |
| --- | --- | --- | --- | --- | --- | --- | --- | --- | --- | --- |
|  |  |  |  |  | **Include AD cases** | | | **Without AD cases** | | |
| **Analysis adjusting for the two SNPs defining the *APOE* alleles** | | | | | | | | | | |
| FinnGen | Finnish | 0.1 | 250kb | 1.00E-06 | 0.95 | 0.05 | 0.28 | 0.89 | 0.06 | 0.08 |
| Jun et al. | Multi-ethnic | 0.2 | 250kb | 1.00E-05 | 1.01 | 0.04 | 0.79 | 0.93 | 0.05 | 0.14 |
| Bellenguez et al. | White | 0.1 | 1000kb | 1.00E-03 | 1.13 | 0.04 | 4.30E-03 | 1.08 | 0.05 | 0.15 |
| Kunkle et al. | White | 0.3 | 1000kb | 1.00E-02 | 1.05 | 0.09 | 0.59 | 0.91 | 0.11 | 0.43 |
| Kunkle et al. | Black | 0.3 | 250kb | 1.00E-04 | 1.02 | 0.02 | 0.46 | 0.99 | 0.03 | 0.73 |
| PRSsum | Multi-ethnic | NA | NA | NA | 1.06 | 0.04 | 0.20 | 0.95 | 0.05 | 0.38 |
| **Analysis without adjusting for the two SNPs defining the *APOE* alleles** | | | | | | | | | | |
| FinnGen | Finnish | 0.1 | 250kb | 1.00E-06 | 1.23 | 0.04 | 2.40E-07 | 1.03 | 0.05 | 0.58 |
| Jun et al. | Multi-ethnic | 0.2 | 250kb | 1.00E-05 | 1.22 | 0.03 | 2.72E-13 | 1.04 | 0.04 | 0.23 |
| Bellenguez et al. | White | 0.1 | 1000kb | 1.00E-03 | 1.22 | 0.04 | 9.81E-07 | 1.11 | 0.05 | 0.04 |
| Kunkle et al. | White | 0.3 | 1000kb | 1.00E-02 | 1.27 | 0.09 | 7.29E-03 | 0.99 | 0.11 | 0.94 |
| Kunkle et al. | Black | 0.3 | 250kb | 1.00E-04 | 1.12 | 0.02 | 1.25E-07 | 1.03 | 0.03 | 0.24 |
| PRSsum | Multi-ethnic | NA | NA | NA | 1.27 | 0.03 | 1.06E-15 | 1.07 | 0.04 | 0.10 |

## Supplementary Table 7: Associations of selected PRS with changes in cognitive functions in SOL-INCA

| **Study** | **Trait** | **N** | **BETA[95% CI]** | **P** | **Analysis Type** |
| --- | --- | --- | --- | --- | --- |
| FinnGen | G-Factor  change | 4037 | -0.01[ 0.02;-0.04] | 0.36 | Adjusting for  APOE alleles |
| Jun et al. |  | 4037 | -0.02[ 0.01;-0.06] | 0.18 |  |
| Bellenguez et al. |  | 4037 | -0.02[ 0.01;-0.04] | 0.24 |  |
| Kunkle et al. 2019 |  | 4037 | -0.01[ 0.05;-0.07] | 0.71 |  |
| Kunkle et al.2021 |  | 4037 | -0.01[ 0.01;-0.04] | 0.35 |  |
| PRSsum |  | 4037 | -0.03[ 0.00;-0.06] | 0.09 |  |
| FinnGen | Digits symbol substitution  change | 4097 | 0.02[ 0.28;-0.24] | 0.88 |  |
| Jun et al. |  | 4097 | -0.05[ 0.23;-0.34] | 0.73 |  |
| Bellenguez et al. |  | 4097 | -0.05[ 0.18;-0.28] | 0.69 |  |
| Kunkle et al. 2019 |  | 4097 | 0.13[ 0.66;-0.41] | 0.65 |  |
| Kunkle et al.2021 |  | 4097 | 0.04[ 0.28;-0.21] | 0.77 |  |
| PRSsum |  | 4097 | -0.01[ 0.28;-0.29] | 0.97 |  |
| FinnGen | SEVLT recall  change | 4198 | -0.05[ 0.05;-0.15] | 0.35 |  |
| Jun et al. |  | 4198 | -0.07[ 0.04;-0.19] | 0.20 |  |
| Bellenguez et al. |  | 4198 | -0.06[ 0.03;-0.15] | 0.19 |  |
| Kunkle et al. 2019 |  | 4198 | -0.16[ 0.05;-0.37] | 0.14 |  |
| Kunkle et al.2021 |  | 4198 | -0.08[ 0.02;-0.18] | 0.11 |  |
| PRSsum |  | 4198 | -0.13[-0.02;-0.24] | 0.03 |  |
| FinnGen | Word frequency  change | 4149 | -0.04[ 0.15;-0.23] | 0.69 |  |
| Jun et al. |  | 4149 | -0.06[ 0.15;-0.27] | 0.57 |  |
| Bellenguez et al. |  | 4149 | -0.03[ 0.14;-0.19] | 0.77 |  |
| Kunkle et al. 2019 |  | 4149 | 0.11[ 0.50;-0.29] | 0.60 |  |
| Kunkle et al.2021 |  | 4149 | -0.02[ 0.16;-0.20] | 0.82 |  |
| PRSsum |  | 4149 | -0.05[ 0.16;-0.26] | 0.65 |  |
| FinnGen | G-Factor  change | 4435 | -0.02[ 0.00;-0.05] | 0.07 | Without adjusting for APOE alleles |
| Jun et al. |  | 4435 | -0.03[-0.01;-0.06] | 0.01 |  |
| Bellenguez et al. |  | 4435 | -0.02[ 0.01;-0.04] | 0.12 |  |
| Kunkle et al. 2019 |  | 4435 | -0.03[ 0.03;-0.09] | 0.32 |  |
| Kunkle et al.2021 |  | 4435 | -0.02[ 0.00;-0.05] | 0.10 |  |
| PRSsum |  | 4435 | -0.04[-0.01;-0.06] | 4.15E-03 |  |
| FinnGen | Digits symbol  change | 4501 | -0.08[ 0.15;-0.30] | 0.49 |  |
| Jun et al. |  | 4501 | -0.22[-0.01;-0.43] | 0.04 |  |
| Bellenguez et al. |  | 4501 | -0.14[ 0.08;-0.36] | 0.20 |  |
| Kunkle et al. 2019 |  | 4501 | -0.04[ 0.48;-0.55] | 0.89 |  |
| Kunkle et al.2021 |  | 4501 | -0.08[ 0.13;-0.30] | 0.46 |  |
| PRSsum |  | 4501 | -0.20[ 0.03;-0.43] | 0.09 |  |
| FinnGen | SEVLT recall  change | 4613 | -0.07[ 0.02;-0.16] | 0.11 |  |
| Jun et al. |  | 4613 | -0.09[-0.01;-0.17] | 0.04 |  |
| Bellenguez et al. |  | 4613 | -0.06[ 0.02;-0.15] | 0.16 |  |
| Kunkle et al. 2019 |  | 4613 | -0.20[ 0.00;-0.40] | 0.05 |  |
| Kunkle et al.2021 |  | 4613 | -0.08[ 0.00;-0.17] | 0.05 |  |
| PRSsum |  | 4613 | -0.13[-0.04;-0.22] | 0.01 |  |
| FinnGen | Word frequency change | 4557 | -0.08[ 0.09;-0.24] | 0.36 |  |
| Jun et al. |  | 4557 | -0.09[ 0.07;-0.25] | 0.26 |  |
| Bellenguez et al. |  | 4557 | -0.03[ 0.13;-0.19] | 0.72 |  |
| Kunkle et al. 2019 |  | 4557 | 0.07[ 0.45;-0.30] | 0.71 |  |
| Kunkle et al.2021 |  | 4557 | -0.03[ 0.12;-0.19] | 0.68 |  |
| PRSsum |  | 4557 | -0.08[ 0.09;-0.25] | 0.35 |  |

# References

[1] P.D. Sorlie, L.M. Avilés-Santa, S. Wassertheil-Smoller, R.C. Kaplan, M.L. Daviglus, A.L. Giachello, et al., Design and implementation of the Hispanic Community Health Study/Study of Latinos., Ann Epidemiol. 20 (2010) 629–641. doi:10.1016/j.annepidem.2010.03.015.

[2] L.M. Lavange, W.D. Kalsbeek, P.D. Sorlie, L.M. Avilés-Santa, R.C. Kaplan, J. Barnhart, et al., Sample design and cohort selection in the Hispanic Community Health Study/Study of Latinos., Ann Epidemiol. 20 (2010) 642–649. doi:10.1016/j.annepidem.2010.05.006.

[3] H.M. González, D. Mungas, B.R. Reed, S. Marshall, M.N. Haan, A new verbal learning and memory test for English- and Spanish-speaking older people., J. Int. Neuropsychol. Soc. 7 (2001) 544–555. doi:10.1017/s1355617701755026.

[4] M.D. Lezak, D.B. Howieson, E.D. Bigler, D. Tranel, Neuropsychological assessment New York: Oxford University Press.[Google Scholar], (2012).

[5] D. Wechsler, WAIS-R manual: Wechsler adult intelligence scale-revised, (1981).

[6] H.M. González, W. Tarraf, M. Fornage, K.A. González, A. Chai, M. Youngblood, et al., A research framework for cognitive aging and Alzheimer’s disease among diverse US Latinos: Design and implementation of the Hispanic Community Health Study/Study of Latinos-Investigation of Neurocognitive Aging (SOL-INCA)., Alzheimers Dement. 15 (2019) 1624–1632. doi:10.1016/j.jalz.2019.08.192.

[7] M.S. Albert, S.T. DeKosky, D. Dickson, B. Dubois, H.H. Feldman, N.C. Fox, et al., The diagnosis of mild cognitive impairment due to Alzheimer’s disease: recommendations from the National Institute on Aging-Alzheimer’s Association workgroups on diagnostic guidelines for Alzheimer’s disease., Alzheimers Dement. 7 (2011) 270–279. doi:10.1016/j.jalz.2011.03.008.

[8] H.M. González, W. Tarraf, N. Schneiderman, M. Fornage, P.M. Vásquez, D. Zeng, et al., Prevalence and correlates of mild cognitive impairment among diverse Hispanics/Latinos: Study of Latinos-Investigation of Neurocognitive Aging results., Alzheimers Dement. 15 (2019) 1507–1515. doi:10.1016/j.jalz.2019.08.202.

[9] M.P. Conomos, C.A. Laurie, A.M. Stilp, S.M. Gogarten, C.P. McHugh, S.C. Nelson, et al., Genetic diversity and association studies in US hispanic/latino populations: applications in the hispanic community health study/study of latinos., Am. J. Hum. Genet. 98 (2016) 165–184. doi:10.1016/j.ajhg.2015.12.001.

[10] M.H. Kowalski, H. Qian, Z. Hou, J.D. Rosen, A.L. Tapia, Y. Shan, et al., Use of >100,000 NHLBI Trans-Omics for Precision Medicine (TOPMed) Consortium whole genome sequences improves imputation quality and detection of rare variant associations in admixed African and Hispanic/Latino populations., PLoS Genet. 15 (2019) e1008500. doi:10.1371/journal.pgen.1008500.

[11] H.M. González, W. Tarraf, X. Jian, P.M. Vásquez, R. Kaplan, B. Thyagarajan, et al., Apolipoprotein E genotypes among diverse middle-aged and older Latinos: Study of Latinos-Investigation of Neurocognitive Aging results (HCHS/SOL)., Sci. Rep. 8 (2018) 17578. doi:10.1038/s41598-018-35573-3.

[12] T. Sofer, J.R. Shaffer, M. Graff, Q. Qi, A.M. Stilp, S.M. Gogarten, et al., Meta-Analysis of Genome-Wide Association Studies with Correlated Individuals: Application to the Hispanic Community Health Study/Study of Latinos (HCHS/SOL)., Genet. Epidemiol. 40 (2016) 492–501. doi:10.1002/gepi.21981.

[13] B.W. Kunkle, B. Grenier-Boley, R. Sims, J.C. Bis, V. Damotte, A.C. Naj, et al., Genetic meta-analysis of diagnosed Alzheimer’s disease identifies new risk loci and implicates Aβ, tau, immunity and lipid processing., Nat. Genet. 51 (2019) 414–430. doi:10.1038/s41588-019-0358-2.

[14] I.E. Jansen, J.E. Savage, K. Watanabe, J. Bryois, D.M. Williams, S. Steinberg, et al., Genome-wide meta-analysis identifies new loci and functional pathways influencing Alzheimer’s disease risk., Nat. Genet. 51 (2019) 404–413. doi:10.1038/s41588-018-0311-9.

[15] E. Granot-Hershkovitz, W. Tarraf, N. Kurniansyah, M. Daviglus, C. Isasi, R. Kaplan, et al., APOE alleles association with neurocognitive function differ across Hispanic background groups, (2020).
